# Supplementary material for: Longitudinal observational cohort study: Speech for Intelligent cognition change tracking and DEtection of Alzheimer’s Disease (SIDE-AD)
Source: BMJ Open. 2024 Mar 28;14(3):e082388. doi: 10.1136/bmjopen-2023-082388 (PMC10982798; doi:10.1136/bmjopen-2023-082388)
Supplement: Supplementary data [file bmjopen-2023-082388supp001.pdf]

Supplemental material
BMJ Publishing Group Limited (BMJ) disclaims all liability and responsibility arising from any reliance placed on this supplemental material which has been supplied by the author(s)
BMJ Open

THE UNIVERSITY of EDINBURGH

NHS Lothian

Participant Information Sheet

Speech for Intelligent cognition change tracking and DEtection of Alzheimer's Disease Research Program: SIDE-AD

Chief Investigator:
Saturnino Luz, PhD

Research team address:
University of Edinburgh
Usher Institute of Public Health
9 Little France Road
Edinburgh BioQuarter
EH16 4UX
side-ad@ed.ac.uk

You are being invited to take part in a research study. Before you decide whether or not to take part, please take time to read the following information carefully. Talk to your family about the study if you wish.

What is the purpose of the study?

Each year we understand more about brain health and how this relates to dementia. Although the brain changes for all of us as we age, and many changes are normal, abnormal changes can be due to an underlying brain disease. One of the most common brain diseases is Alzheimer's disease, which causes dementia.

We now know that when Alzheimer's disease develops, there are changes in the brain which may occur many years before memory and thinking skills are affected. This is typically many years before somebody is ultimately diagnosed with dementia. Throughout this information sheet, when we talk about Alzheimer's disease, we mean the underlying brain disease that over many years may lead to Alzheimer's dementia. Currently, doctors can only diagnose Alzheimer's disease at the most advanced stage when people already have dementia or are very close to developing it.

The purpose of this study is to understand more about speech as potential early indicator of dementia risk. This is because changes in how somebody speaks may show markers of pathological changes in Alzheimer's disease. The SIDE-AD study is a

Saunders S, et al. BMJ Open 2024; 14:e082388. doi: 10.1136/bmjopen-2023-082388

longitudinal cohort study which means that if you take part in the study, you will be emailed a follow-up email to carry out follow-up assessments. Taking part in the study and in the follow-up assessments is all voluntary.

This study aims to develop digital speech-based markers for Alzheimer's disease. Please note that the speech assessment is not a diagnostic tool for Alzheimer's disease but is used to study the disease.

Why have I been invited to take part?

You have been approached because you are either healthy and do not experience cognitive decline or you are engaged with secondary services for cognitive decline and may or may not have a diagnosis related to memory concerns.

We invite individuals through the following paths:

- Taking part in the PREVENT cohort study
- Being a member of a research register
- Attending NHS memory assessment services

We aim to recruit 350 participants from the PREVENT cohort study and research registers and 150 participants from NHS services.

To find out which factors may predict risk of Alzheimer's dementia, we need a broad range of people with different likelihoods of developing Alzheimer's dementia. We do not know in advance who will develop dementia and who will not. This means we aim to include in the SIDE-AD study people who are possibly at high risk, low risk and anywhere in between. As we follow all the people in the SIDE-AD study, we learn which factors predict risk of dementia. Only by including a wide range of people can we identify risk factors Alzheimer's dementia.

Research team address:
University of Edinburgh
Usher Institute of Public Health
9 Little France Road
Edinburgh BioQuarter
EH16 4UX; side-ad@ed.ac.uk

Saunders S, *et al. BMJ Open* 2024; 14:e082388. doi: 10.1136/bmjopen-2023-082388

Do I have to take part?

No, it is up to you to decide whether or not to take part. If you decide to take part, you are still free to withdraw at any time and without giving a reason. Deciding not to take part or withdrawing from the study will not affect the clinical care that you receive.

What will happen if I take part?

This is a longitudinal study. Longitudinal simply means that we will send out emails for follow-up assessments every three months over two years. All assessments are done remotely at home, and you are free to withdraw at any point. Each study assessment will take about 5 min and you are asked to use either your computer or any smart device. A study assessment includes:

- *Background information about you*
- *Four questions about your mood, anxiety and apathy levels*
- *Recording yourself speak for about a minute for speech analysis*
  You could talk about any aspects about brain health. For example, you could talk about what brain health means to you, what you do to look after your brain health or what you feel you could do differently.

If you decide to take part in the study, please go to the below website:

tiny.cc/speechstudy

Before you start the study, we ask that you read this information sheet and consent to taking part in the study. Please read the statements carefully and write your initials in the box if you agree. If you have any questions about the study, please contact the research team. After you have given electronic consent to taking part in the study, the assessments will start.

Research team address:
University of Edinburgh
Usher Institute of Public Health
9 Little France Road
Edinburgh BioQuarter
EH16 4UX; side-ad@ed.ac.uk

Saunders S, *et al. BMJ Open* 2024; 14:e082388. doi: 10.1136/bmjopen-2023-082388

Please note that on the consent form, we will also ask for your permission to be contacted to discuss record linkage. If you consent to this, a member of our research team will contact you and have an informed discussion with you whether you agree to record linkage and take your consent. Record linkage is applicable for participants who are recruited through clinical services and means that we can match your study data to your health records collected during your routine NHS care. We will do this using your name and date of birth. This can be really useful to researchers to understand what research data means in relation to clinical findings such as brain scans, routine blood tests and treatment decisions or diagnosis. You do not have to agree to this to be in the study.

if you are having problems completing the study activities during the course of the study (meaning you have difficulties understanding information and making a decision based on it), you can still remain in the study unless you decide to leave. This simply means that whether you experience problems with your brain health such as remembering things and planning or you do not experience these kinds of problems, you are welcome to continue taking part in our study. if you are having problems completing the study activities, you are welcome to get help from friends or family with any aspects of the study.

At any stage in the study, if you no longer wish to continue, you can withdraw from either all aspects of the study but allow us to use data collected up to the point where you discontinue **OR** you can withdraw from all aspects of the study with removal of all previously collected data. You simply have to tell us what you prefer. It is important to realise that when you start the study you are free to leave at any time point after giving consent.

Research team address:
University of Edinburgh
Usher Institute of Public Health
9 Little France Road
Edinburgh BioQuarter
EH16 4UX; side-ad@ed.ac.uk

Saunders S, *et al. BMJ Open* 2024; 14:e082388. doi: 10.1136/bmjopen-2023-082388

What are the possible benefits of taking part?

You should not expect to benefit medically from being involved in this project. Participants can find participating in studies like this enjoyable.

Your contribution to this research enables us to further our understanding of Alzheimer's disease. Specifically, taking part in the study may help researchers and clinicians in identifying early signs of Alzheimer's disease and ultimately to treat or prevent dementia.

What are the possible disadvantages of taking part?

We appreciate that taking part in the study assessments is a time commitment and we will do everything we can to fit into your schedule and be as flexible as possible.

The study is entirely remote which means you can choose the time when is suitable for you to do the study assessments.

What if there are any problems?

If you have a concern about any aspect of this study, please contact the study team who will be delighted to answer any questions about the study. If you would like to discuss this study with someone independent of the study, please contact Dr Miles Welstead (Miles.Welstead@ed.ac.uk) who will do his best to answer your questions. However, if you are still unhappy with the answers given and wish to complain, you can do this through the NHS Lothian Patient Experience Team (2nd Floor; Waverley Gate; 2 – 4 Waterloo Place; Edinburgh; EH1 3EG; Tel: 0131 536 3370; email: feedback@nhslothian.scot.nhs.uk).

Will my taking part be kept confidential?

You will be assigned a participant number and you will not be personally identified in any of the research data. Your personal data, including your voice recordings, will not leave the research site. Your voice data will be stored on secure

Research team address:
University of Edinburgh
Usher Institute of Public Health
9 Little France Road
Edinburgh BioQuarter
EH16 4UX; side-ad@ed.ac.uk

University of Edinburgh servers (servers are large storage spaces for computer data and be password protected) and will only be accessible to legitimate members of the research team and is approved by the sponsor. In order to monitor and audit the study we will ask for your consent for responsible representatives from the study sponsor and NHS Institution(s) to access your data collected during the study. The Sponsors are responsible for overall management of the study and providing insurance and indemnity. The sponsor is the University of Edinburgh and NHS Lothian under a joint agreement between those two organisations called ACCORD.

What will happen to the results of the study?

To analyse early indicators of Alzheimer's disease, we pool together data from all the study participants. This means that we do not analyse individual participant trajectories (changes in risk factors over time) but look for patterns across all the study participants.

External researchers will be able to apply to access research data from the SIDE-AD study. Please note that your voice recordings will not be shared. Research data is anonymous, and you will not be identifiable in any of this data. We think this is important to maximise the use of all data collected in the study. Sony Research Awards who funds this study may have access to the anonymised results of this study.

Your voice data may be transcribed by an approved 3rd party vendor. This means that the transcribing company we use will respect the confidentiality of your data and their work has been approved by the University of Edinburgh. Transcribing means writing out all the spoken information you give and we record into text. All data transcripts will be anonymised. We will prepare scientific publications based on the findings from the SIDE-AD study which will be presented at national and international conferences. You will not be identified in any of the publications. We will communicate findings from these studies in regular newsletters, which you can sign up to receive.

Research team address:
University of Edinburgh
Usher Institute of Public Health
9 Little France Road
Edinburgh BioQuarter
EH16 4UX; side-ad@ed.ac.uk

Saunders S, *et al. BMJ Open* 2024; 14:e082388. doi: 10.1136/bmjopen-2023-082388

Where can you find out more about how your information is used?

You can find out more about how we use your information

- at www.hra.nhs.uk/information-about-patients/
- our leaflet available from www.hra.nhs.uk/patientdataandresearch
- by asking one of the research team
- by sending an email to the Sponsor's Data Protection Officer dpo@ed.ac.uk

Who is organising and funding the research?

This study is led by the University of Edinburgh, Dr Saturnino Luz. The other team members include Dr Fasih Haider and Dr Stina Saunders who are both postdoctoral research fellows at the University of Edinburgh. This study is funded by Sony Research Awards.

Who has reviewed the study?

All research in the NHS is looked at by an independent group of people called a Research Ethics Committee. A favourable ethical opinion has been obtained from XXXX. NHS management approval has also been given. IRAS Project ID 323311.

Researcher Contact Details

For any questions concerns, please email Dr Stina Saunders (Stina.Saunders@ed.ac.uk) or dedicated study contact email side-ad@ed.ac.uk.
University of Edinburgh
Usher Institute of Public Health
9 Little France Road
Edinburgh BioQuarter
Edinburgh
EH16 4UX

Independent Contact Details

If you would like to discuss this study with someone independent of the study, please contact Dr Miles Welstead (Miles.Welstead@ed.ac.uk)

Complaints

If you wish to make a complaint about the study please contact:
Patient Experience Team
2 – 4 Waterloo Place, Edinburgh, EH1 3EG
feedback@nhslothian.scot.nhs.uk
0131 536 3370

Thank you for considering your involvement in this project and taking time to read this information sheet

Saunders S, *et al. BMJ Open* 2024; 14:e082388. doi: 10.1136/bmjopen-2023-082388
