# Supplementary material for: Longitudinal observational cohort study: Speech for Intelligent cognition change tracking and DEtection of Alzheimer’s Disease (SIDE-AD)
Source: BMJ Open. 2024 Mar 28;14(3):e082388. doi: 10.1136/bmjopen-2023-082388 (PMC10982798; doi:10.1136/bmjopen-2023-082388)
Supplement: Supplementary data [file bmjopen-2023-082388supp002.pdf]

THE UNIVERSITY of EDINBURGH

NHS Lothian

Electronic Informed Consent Form for Participants

Speech for Intelligent cognition change tracking and DEtection of AD (SIDE-AD) Research Program: SIDE-AD

This document is for review purposes only, consent takes place electronically using the following link tiny.cc/speechstudy

	Enter your Initials (electronic)
1. I confirm that I have read and understand the information sheet (SIDE-AD PIS v4 29 Nov 2023) for the above study. I have had the opportunity to consider the information, ask questions and have had these questions answered satisfactorily.	
2. I understand that my participation is voluntary and that I am free to withdraw at any time without giving any reason and without my medical care and/or legal rights being affected.	
3. I understand that data collected about me during the study may be converted to anonymised data.	
4. I understand that relevant sections of my data collected during the study may be looked at by individuals from the Sponsor (University of Edinburgh and NHS Lothian under a joint agreement between those two organisations called ACCORD) and/or from regulatory authorities where it is relevant to my taking part in this research. I give permission for these individuals to have access to my data and/or medical records.	
5. I agree to my voice being recorded as part of the study.	
6. I understand that if I lose capacity during the course of this study, I will remain in the current study or I am free to withdraw. This means that whether I experience problems with my brain health or I do not experience problems, I am welcome to continue taking part in this study.	

SIDE-AD ICF v1 24 Jul 2023
IRAS Project ID: 323311

Saunders S, et al. BMJ Open 2024; 14:e082388. doi: 10.1136/bmjopen-2023-082388

7. I agree to my audio recordings being transcribed by a third-party contractor.	
8. I understand that information collected will be managed by the research team only and will be destroyed after a period of ten years and any information collected will be kept in line with the data protection act in a secure way.	
9. I agree to my anonymised data being used in future studies.	
10. I agree to be re-contacted about future research.	
11. I confirm that I am over 40 years of age.	
12. I agree to take part in the above study.	
Final statement is only applicable to individuals recruited through NHS memory clinics. *If you agree to this statement, a member of the research team will contact you to explain this further and ask for your consent to access relevant sections of your Medical Records. Agreeing to this statement* ***does not grant access*** *to your Medical Records. Please enter* ***initials*** *below if you agree.*	
**13. Optional:** I agree to be contacted about the potential to link my study data with my NHS Medical Records.	
Enter Name:	
Date:	
