# Supplementary material for: Longitudinal observational cohort study: Speech for Intelligent cognition change tracking and DEtection of Alzheimer’s Disease (SIDE-AD)
Source: BMJ Open. 2024 Mar 28;14(3):e082388. doi: 10.1136/bmjopen-2023-082388 (PMC10982798; doi:10.1136/bmjopen-2023-082388)
Supplement: Author's manuscript [file bmjopen-2023-082388.draft_revisions.pdf]

BMJ Open

BMJ Open is committed to open peer review. As part of this commitment we make the peer review history of every article we publish publicly available.

When an article is published we post the peer reviewers' comments and the authors' responses online. We also post the versions of the paper that were used during peer review. These are the versions that the peer review comments apply to.

The versions of the paper that follow are the versions that were submitted during the peer review process. They are not the versions of record or the final published versions. They should not be cited or distributed as the published version of this manuscript.

BMJ Open is an open access journal and the full, final, typeset and author-corrected version of record of the manuscript is available on our site with no access controls, subscription charges or pay-per-view fees (http://bmjopen.bmj.com).

If you have any questions on BMJ Open's open peer review process please email info.bmjopen@bmj.com

BMJ Open

Protocol: Longitudinal observational cohort study: Speech for Intelligent cognition change tracking and DEtection of AD (SIDE-AD)

Journal:	*BMJ Open*
Manuscript ID	bmjopen-2023-082388
Article Type:	Protocol
Date Submitted by the Author:	22-Nov-2023
Complete List of Authors:	Saunders, Stina; University of Edinburgh<br>Haider, Fasih; University of Edinburgh, Usher Institute<br>Ritchie, Craig; Centre for Clinical Brain Sciences, Department of Psychiatry, University of Edinburgh; Scottish Brain Sciences<br>Muniz Terrera, Graciela; University of Edinburgh, Centre for Dementia Prevention; Ohio University, Heritage College of Osteopathic Medicine<br>Luz, Saturnino; University of Edinburgh School of Molecular Genetic and Population Health Sciences, Usher Institute of Population Health Sciences and Informatics
Keywords:	NEUROLOGY, PUBLIC HEALTH, Clinical Trial

SCHOLARONE™
Manuscripts

Protocol: Longitudinal observational cohort study: Speech for Intelligent cognition change tracking and DEtection of AD (SIDE-AD)

Saunders, S.[1] (PhD), Haider, F. (PhD)[1], Ritchie, C.W.[2,3] (MD PhD), Muniz-Terrera, G.[3,4] (PhD), Luz, S.[1] (PhD)

[1]*Usher Institute; Edinburgh Medical School, University of Edinburgh, Edinburgh, UK*

[2] *Scottish Brain Sciences, Edinburgh, UK*

[3]*Centre for Clinical Brain Sciences, University of Edinburgh, Edinburgh, UK*

[4]*Ohio University, Heritage College of Osteopathic Medicine, Ohio, US*

Corresponding author: Stina Saunders (Stina.Saunders@ed.ac.uk)

Stina Saunders
Usher Institute
University of Edinburgh

Declarations of interest: none (all authors)

Word count 3,888

For peer review only

ABSTRACT

Introduction

There is emerging evidence that speech may be a potential indicator and manifestation of early Alzheimer's disease (AD) pathology. Therefore, the University of Edinburgh and Sony Research have partnered to create the SIDE-AD (Speech for Intelligent cognition change tracking and DEtection of Alzheimer's Disease) study which aims to develop digital speech-based biomarkers for use in neurodegenerative disease.

Methods and Analysis

SIDE-AD is an observational longitudinal study, collecting samples of spontaneous speech. Participants are recruited from existing cohort studies as well as NHS memory clinics in Scotland. Using an online platform, participants record a voice sample talking about their brain health and rate their mood, anxiety and apathy. The speech biomarkers will be analysed longitudinally, and we will use machine learning and natural language processing technology to automate the assessment of the respondents' speech patterns.

Ethics and Dissemination

The SIDE-AD study has been approved by the NHS Research Ethics Committee (REC reference: 23/WM/0153, protocol number AC23046, IRAS Project ID 323311) and received NHS management approvals from Lothian, Fife and Forth Valley NHS boards. Our main ethical considerations pertain to the remote administration of the study, such as taking remote consent. To address this, we implemented a consent process whereby the first step of the consent is done entirely remotely but a member of the research team contacts the participant over the phone to consent participants to the most sensitive, optional, elements of the study. Results will be presented at conferences, published in peer reviewed journals, and communicated to study participants.

Keywords: speech analysis; Alzheimer's disease; early detection

For peer review only - http://bmjopen.bmj.com/site/about/guidelines.xhtml

ARTICLE SUMMARY

Strengths and limitations of this study

- The main strength of this study is that it combines assessment of cognition with mood, anxiety and apathy assessment over time, through spontaneous speech which can be collected remotely, unobtrusively and at scale.
- Another strength is its ability to relate patterns of speech collected while the participant is talking about their brain health to cognitive, clinical and functional outcomes through existing cohort data, and NHS patient record data (optional consent).
- Assessment by analysis of spontaneous speech, by relying on an inherently repeatable task that is not prone to practice effects, provides a way of avoiding variability in cognitive assessment due to extraneous environmental or subjective factors which could distort the true trajectory of cognitive performance over time.
- A limitation of the study is that there is no way to control for variability in audio recording equipment, as participants will be using their own personal computers or mobile phones to collect the speech samples. It is also possible the recording might contain background noise and might be affected by reverberation in the recoding room.
- Another possible limitation is the heterogeneity of data between the recruitment sources, an existing longitudinal cohort study and the NHS. Although the study will collect a standardised self-assessment baseline, the differences in background cohort and records data complicates the analysis.

INTRODUCTION

Dementia is a major public health concern with over 55 million people currently living with dementia worldwide and recent projections reaching 78 million living with dementia by 2030 (Gauthier et al. 2021). Alzheimer's disease (AD) is the most common underlying pathology for the dementia syndrome, accounting for 60 to 70% of all dementia cases (Ballardsych et al., 2011; Silva et al., 2019) and is also one of the leading causes of death in the US (Ahmad et al., 2021). Due to a long preclinical period in the AD pathology, dementia is a late-stage manifestation of illness that has been developing years before dementia manifests (DeTure et al., 2019; McDade et al., 2018). Though not yet globally available, recent pharmacological advances have yielded several new medications for the treatment of AD (e.g., Haeberlein et al., 2022; Rashad et al., 2022) and there are many other pharmacological approaches in progress which necessitate detection of AD in its earliest pathological stage.

Dementia is an illness that causes neurodegeneration whereby the brain gradually transitions from a healthy brain to brain failure (Golde, 2022). In the initial symptomatic stages, deterioration of brain health manifests as cognitive impairment, though over time once dementia develops, the cognitive impairment cascades into problems with daily functioning. Additionally, symptomatic AD may change an

For peer review only - http://bmjopen.bmj.com/site/about/guidelines.xhtml

individual's language, especially certain elements in speech such as elaboration (e.g., making an argument clearer or providing examples) and attribution (e.g., the ability to attribute characteristics to others) (Abdalla et al., 2018). What is more, evidence suggests that there is measurable difference between the speech patterns for individuals who are ageing with and without cognitive decline. For example, a recent systematic review concluded that compared to healthy individuals, those with AD had a decline in the amount of meaningful content expressed, reduction of total output of expressive language and a preference for generic verbs and simple syntax (Williams et al., 2021). The same review also concluded that people with AD had greater difficulties comprehending sentences featuring multiple verbs or verbs with reversible thematic roles than individuals who did not have AD.

Currently, the primary outcomes used in AD clinical trials focus on cognition and biomarker data (e.g., brain imaging, analysing cerebrospinal fluid or blood tests) (Posner et al., 2017), there is emerging evidence that speech analysis could become a promising tool for detecting early neurodegenerative disease (Vrahatis et al., 2023). A systematic review of studies looking to distinguish healthy ageing from cognitive decline in older adults using speech features concluded that most studies record a diagnostic accuracy over 88% for AD and 80% for MCI (Martínez-Nicolás et al., 2021). Furthermore, a study by Eyigoz et al. (2020) predicted future diagnosis of AD among a cognitively normal baseline of Framingham Heart Study participants and demonstrated that linguistic features from a single administration of the cookie-theft picture description task out-performed predictive models that incorporated genetic risk status, demographic variables, and neuropsychological test results.

In our own systematic review on the potential of using interactive AI methods to detect AD biomarkers, we concluded that when compared to traditional neuropsychological assessment methods, speech and language technology were at least equally discriminative between different groups (de la Fuente et al., 2020). Interestingly, speech-based markers of AD pathology may be confounded by some of AD's core pathology as people with higher amyloid levels (both asymptomatic and those with mild cognitive impairment) exhibit a greater rate of decline in episodic memory and language (Lim et al., 2014). While research on speech biomarkers is only beginning to emerge, we expect speech and language analysis to play a role in detecting and tracking preclinical AD in the near future (Öhman et al., 2021; Luz et al., 2021).

In addition to cognition, speech and language are indicators of neurobehavioral outcomes such as mood, apathy and anxiety, and have been used as input data in machine learning based emotion recognition models (de Lope and Graña, 2023). Automatic methods for assessment of mood, anxiety and apathy could be an important component in AD detection and on-going monitoring of patient outcomes. Apathy is the most common neuropsychiatric symptom in AD patients, and its accurate detection is important in terms of treatment outcomes, and carer education

For peer review only - http://bmjopen.bmj.com/site/about/guidelines.xhtml

and well-being (Landes et al., 2005). Furthermore, mood disorders have also been linked to AD pathology (Babulal et al., 2016).

To date, most studies of neurodegenerative diseases involving speech have been laboratory-based, and rarely included spontaneous speech. However, there are advantages in collecting spontaneous connected speech. Spontaneous speech can be captured in more natural settings over time, thus mitigating problems that might affect performance in controlled, cross-sectional data, such as a participant having an "off day" or having poorly slept the night before the test. Speech can also be collected remotely, using widespread mobile devices and smartphones, which facilitates data collection at scale and over time (Luz, 2017). Early recognition of individuals at risk of AD is important in order to intervene at a time where any intervention may yield the most benefit.

This study explores changes in speech as potential early markers of pathological changes in AD. The SIDE-AD study is a longitudinal cohort study of individuals across the AD risk spectrum, those who are healthy volunteers and those engaged with secondary services for cognitive decline.

The study aims to develop digital speech-based biomarkers for AD in order to assess disease status in the pre-dementia stages in a real-world population.

METHODS AND ANALYSIS

We use the Standard Protocol Items for Randomized Trials (SPIRIT) reporting guidelines (Chan et al., 2013) for the SIDE-AD study.

This is a remote observational longitudinal study developing speech biomarkers for use in neurodegenerative disease. The SIDE-AD study involves collecting voice data using an online platform and prompting individuals to talk about brain health (Figure 1) and the study has started on protocol version 3, dated 20 Sep 2023.

Figure 1. Speech for Intelligent cognition change tracking and DEtection of AD (SIDE-AD) study flow

How the sample will be selected

The selection of the participant population aims to create a risk spectrum of individuals with a low to high risk of AD. Therefore, the participant population includes individuals taking part in a longitudinal cohort study aimed at dementia prevention who are healthy volunteers but may have risk factors for dementia (such as genetic risk, increased amyloid or objective cognitive decline) and patients referred to clinical services who present with concerns around cognitive decline in NHS memory clinics in the UK.

Participants will be identified through two different routes:

For peer review only - http://bmjopen.bmj.com/site/about/guidelines.xhtml

1. Participants taking part in the PREVENT Dementia study (Ritchie and Ritchie, 2012)

The PREVENT study is a longitudinal cohort study of individuals in mid-life with n=700 participants, creating a spectrum of individuals from low to high risk of dementia. The Participant Information Sheet (PIS) will be emailed by the PREVENT study team.

2. Participants will be identified through NHS memory assessment services

Individuals will be either emailed the PIS electronically (Appendix 1) or handed the study PIS during routine clinic visits.

In order to achieve a spectrum of low to high individuals at risk of AD, additional recruitment sources may be added to the SIDE-AD study through protocol amendments. Protocol amendments will be communicated to relevant parties in line with regulatory requirements.

Eligibility

INCLUSION CRITERIA

- Over 40 years old
- Ability to carry out remote assessments (access to a computer/smart device)
- Have the capacity to consent
- Participating in the PREVENT study or attending an NHS memory clinic

EXCLUSION CRITERIA

- Not able to speak English
- Individuals who have not provided consent

Sample size calculation

We aim to recruit a total of 450 participants over a 10-month period (Oct 2023-Jun 2024). The PREVENT study has 700 participants overall and at the first stage, only participants from Scotland (n=300) will be invited to take part in the SIDE-AD study. Additionally, we estimate the number of eligible individuals in NHS clinical settings to be around 150 over 10 months which will be sufficient for our planned analyses.

There is no a priori sample size calculation as the SIDE-AD study will recruit from the PREVENT study and enrich the sample with a clinical population recruiting from NHS memory clinics where all eligible individuals will be invited to take part in the SIDE-AD study. It is difficult to estimate precisely the optimal sample sizes and measurable effect sizes for machine-learning based studies such as SIDE-AD. In the field of medical decision support systems, a commonly adopted rule of thumb for determining sample size in continuous outcome settings is to require two participants per feature (predictor) in the model. While this heuristic approach has been criticised for not

For peer review only - http://bmjopen.bmj.com/site/about/guidelines.xhtml

taking into account the context of different studies (Riley et al., 2020), some theoretical justification for it has been offered in the pattern recognition literature for the case of classification (Raudys and Jain, 1991). According to this approach, for relatively simple machine learning algorithms such as Euclidean distance and linear discriminant analysis, lower bounds at 1.2 and 1.4 participants per feature has been proposed, for an expected probability of misclassification (PMC) at most 50% greater than an asymptotic PMC of 0.1. Using the extended Geneva Minimalistic Acoustic Parameter Set (eGeMAPs) feature set (Eyben et al., 2016) as proposed here, would therefore imply a minimum of 114 participants for a PMC at most 50% greater than a conservative asymptotic PMC of 0.1 (90% accuracy). However, this estimate presupposes the use of a fairly simple linear model. We aim to collect data from larger numbers of participants, which will allow us to experiment with larger sets of speech features. In the Riley et al. (2020) paper, a method is proposed for estimation of sample sizes for continuous outcomes regression models that aims to ensure that the data sample is large enough to estimate the model intercept and residual variance precisely, and account for the risk of overfitting by setting adequate regularisation and optimism targets. While this approach only covers standard regression models, it provides more conservative sample size estimates. Taking a previous study on anxiety and depression recognition from speech conducted by our group (De la Fuente et al., 2021) as a basis, and assuming a model R2 of 0.3, a regularisation factor of 0.8, mean arousal and valence scores of 30, standard deviation of 22 and a reduced feature set of 40 would require sample size of 421 participants, according to the formula given in Riley et al. (2020). It is speculated in the Riley et al. (2020) paper that machine learning models require much larger sample size than simpler statistical models. We intend to use existing datasets containing speech samples from over 1,000 subjects for data augmentation and dimensionality reduction methods, such as Haider et al. (2019), should our study's sample sizes prove insufficient to train accurate predictive models.

What outcomes will be measured

As this study aims to develop models for prediction of AD and related neurobehavioural outcomes, the primary endpoints of this study are measures of model prediction performance, namely, root mean squared error (RMSE) and coefficient of determination values for predicted cognitive scores, and mood, anxiety and apathy prediction ratings, for regression models, and area under the receiver operating characteristic curve (AUROC), sensitivity and specificity for classification into score categories (e.g. high vs low mood etc).

In terms of secondary endpoints, all participants in the SIDE-AD study will self-report the following outcomes which are used as explanatory variables (Table 1).

Variables collected as part of SIDE-AD study (all participants)	
**Spontaneous speech**	Voice-recording in response to: "Please tell us in your own words how you look

For peer review only - http://bmjopen.bmj.com/site/about/guidelines.xhtml

	after your brain health.”
**Sociodemographic details**	Date of birth<br>Gender<br>Education
**Self-reported neurodegenerative disease diagnosis**	Mild Cognitive Impairment<br>Alzheimer’s disease<br>Mixed dementia<br>Dementia with Lewy bodies<br>Frontotemporal dementia<br>Vascular dementia<br>Parkinson’s disease dementia<br>Parkinson’s disease<br>Huntington’s disease<br>Creutzfeldt-Jakob Disease<br>Korsakoff Syndrome<br>Motor neuron disease (also known as ALS or Amyotrophic lateral sclerosis)<br>Subjective Cognitive Disorder<br>Major neurocognitive disorder<br>Stroke
**Self-reported mood**	1-100 score on arousal and happiness
**Self-reported anxiety**	1-100 score
**Self-reported apathy**	1-100 score

Table 1 Variables collected as part of SIDE-AD study

For participants recruited through the PREVENT Dementia study, SIDE-AD will have access to the following variables collected as part of the PREVENT protocol:

- Demographics (Date of Birth; Gender; Race; Education)
- Biosamples (APOE e4 status, amyloid status)
- Risk Factors (self-reported memory concerns and health status, being out of breath, family history of dementia)
- Lifestyle Interview (Smoking status, Electronic cigarette use, Drug use, Alcohol Intake, Diet)
- Cognitive Outcomes
- National Adult Reading Test (NARTs)
- Visual Short Term Memory Binding Test
- Cognito
- ACE- III

For peer review only - http://bmjopen.bmj.com/site/about/guidelines.xhtml

- Supermarket Task
- 4 Mountains Task

- Clinical and Functional Outcomes
- Apathy Inventory
- Lawton Instrumental Activities Of Daily Life Scale
- CES-D Depression Scale
- Spielberger State Scale
- Pittsburgh Sleep Quality Index
- Sleep Questionnaire
- Epworth Sleepiness Scale
- Life Stressor Checklist
- Brain Injury Screening Questionnaire

- Dementia Risk Score
- CAIDE score

Individuals who are recruited through NHS services will have an optional consent for record linkage. For those participants who are recruited through NHS services and who consent to record linkage, changes in speech will be associated with routinely collected health care data.

Record linkage will include any/all the available outcomes:

- Neurodegenerative disease diagnosis
- Clinical outcomes (ADL, mood/anxiety and sleep measures)
- Neuropsychological outcomes (cognitive tests)
- Biomarker outcomes: Genetic risk (ApoE4)
- Biomarker outcomes: Amyloid levels
- Biomarker outcomes: Tau levels
- Neuroimaging outcomes

When and how

The SIDE-AD study is self-administered remotely, though a member of the research team is available via contact details provided on the study PIS and study website. The assessment takes around 5 minutes to complete on a computer or smart device and the participant receives an email invitation for follow-up assessments every 3 months.

Interested participants follow a unique URL link to the SIDE-AD research platform, where they will be asked to give consent (Appendix 2) and perform the baseline assessment. This is done remotely from the participants' home with no direct involvement of the research team. All individuals in the SIDE-AD study will be invited

For peer review only

to provide speech samples through the online platform. Assessments, including speech sampling, will be done quarterly over the study duration.

Participant retention and completion of follow-up is promoted through reminders in each follow-up invitation that participants' contribution is valued and voluntary.

Data analysis plan

We will conduct descriptive analysis of the sociodemographic information provided by the individuals, using simple tabulations. A lay language report with the survey results from the analysis of the data will be produced and made publicly available.

We developed the SIDE-AD platform to collect speech data and limited background/self-reported mood, anxiety and apathy data. The platform is used to monitor speech patterns over time. We will extract speech markers and evaluating a comprehensive set of acoustic features for modelling and prediction of cognitive function.

We will use the primary data for the purpose of developing models for the assessment of cognition, mood, anxiety and apathy. We will extract the following speech features and their most common statistical functionals (in-total, 88 low-level descriptors of the enhanced Geneva Minimalistic Acoustic Parameter Set [eGeMAP] as per Eyben et al., 2016) from the collected speech signal.

- F0 semitone: F0 is determined physiologically by the number of cycles that the vocal folds make in a second, corresponding to prosodic aspects of speech;
- Loudness: the loudness of speech implies an increase in the vocal effort which describes a strained or tense voice quality;
- Spectral flux: a low-level feature that measures how quickly the power spectrum of a signal changes, used for voice activity detection;
- Mel-frequency cepstral coefficients (MFCC):represent spectral information from the speech signal, using a scale known as the "Mel-frequency scale," which is chosen to mimic the way humans perceive audio;
- Jitter: this indicates the variability or perturbation of F0 (i.e. a measure of frequency instability)
- Shimmer: this is also also a measures of F0 perturbation but is related to the amplitude of the sound wave or the intensity of vocal emission (i.e. a measure of amplitude instability). A normal healthy voice has a small amount of instability during sustained vowel production, and tissue and muscle properties influence normal instabilities.
- F1, F2, F3: these are formant frequencies that relate to characteristics of the speaker's vocal tract;
- Alpha ratio: the ratio between the energy in the low-frequency region and the high-frequency region; a measure correlated to perceived voice quality;

For peer review only - http://bmjopen.bmj.com/site/about/guidelines.xhtml

- Hammarberg index: a spectral measure used as an energy distribution measure averaged across an utterance;
- Spectral Slope (linear regression within bands 0-500Hz and 500-1500Hz): a feature that has been investigated in connection with emotional speech.

Once these features have been extracted. we will apply the Active Data Representation (ADR) method (Haider et al., 2019) for dimensionality reduction, in order to extract the relevant dimensions of variation of the signal, while downplaying contingent voice features. Finally, we will train support vector regression models for automatic prediction of cognitive status (cognitive test scores), and mood, anxiety, and apathy levels (self-reported). We will also explore the relationship between these inferred and self-reported neurobehavioral outcomes and cognitive function through Pearson correlation statistical test and through machine learning modelling, with mood, anxiety and apathy used as additional input features for the model, along with speech features. Where possible, we will discretise the cognitive and neurobehavioral outcomes (mood ratings can be assigned to three classes: depressed, healthy, and (hypo)manic) and train classification models to predict the corresponding classes. Performance of regression models will be assessed throughout the root mean squared error (RMSE) metric and the coefficient of determination. Performance of classification models will be assessed by computing the area under the receiver operating characteristic (ROC) curve, and sensitivity and specificity metrics.

ETHICS AND DISSEMINATION: ETHICAL AND SAFETY CONSIDERATIONS

We consider the main issues to pertain to the remote administration of the study. We have included contact details of the research team on every page of the survey and invite individuals to contact the team should they have any questions after reading the participant information sheet accompanying the informed consent form. We have also engaged with the study sponsor Academic and Clinical Central Office for Research and Development (ACCORD; resgov@accord.scot) and the NHS information governance and IT security teams from the initial design stages of the study protocol to ensure remote consent is a secure method for consent. Our solution was to implement a two-stage consent process whereby the first step of the consent is done entirely remotely but the participant has an option to consent to being contacted about linking to relevant sections in their memory clinic records. If participants consent to being contacted about record linkage, a member of the SIDE-AD study research team contacts the participant and while the second step of the optional record linkage is electronic, it is only after a conversation with a member of the research team which also allows the research team to assess capacity to consent. We note the optional record linkage consent only applies to a small number of participants who are recruited through NHS memory clinics.

We considered this to be an appropriate process from both Good Clinical Practice (GCP) and data security point of view, but this was also the favoured method to consent by

For peer review only - http://bmjopen.bmj.com/site/about/guidelines.xhtml

our patient and public involvement (PPI) contributors who gave feedback at the design stage of the protocol/survey. The feedback also considered the study to be fairly low impact only taking a few min to complete which does not necessitate face to face consent.

The other issue we have carefully considered is recording individuals' speech. We remind individuals not to disclose personally identifiable information in the speech recording and we also explain that recording of the speech sample is for research purposes only and not part of clinical care.

THE PATIENT AND PUBLIC INVOLVEMENT STATEMENT

Patients and the public were first involved in the research at the design stage. We will also involve patients and public in the analysis of results and dissemination of findings. For the design stage, we contacted the Neuroprogressive and Dementia Network PPI panel in Scotland for feedback on our survey platform, participant information sheet and consent forms. We received feedback from 10 individuals both healthy volunteers and those with lived experience of neurodegenerative disease. We carefully reviewed feedback and implemented changes proposed by the PPI contributors, these pertained primarily to the online survey platform but also to the information provided about the study. Overall, the PPI input was favourable, and individuals saw value in a remote study developing speech based biomarkers which was perceived to have low participant burden but reward for taking part in research. Some of the issues raised involved giving more guidance on what people could talk about when they are asked to record speech and to add a box for optional comments for if individuals want to add a note about their recording to the research team. We also intend to involve PPI contributors in the analysis of results and dissemination of findings to help prioritise what information to share, at which conferences/workshops and in which format. We plan to do this via the Neuroprogressive and Dementia Network; EPAD participant panel or National Dementia Carers Action Network (part of Alzheimer Scotland).

DISSEMINATION PLAN

A possible dissemination opportunity will be the Scottish Dementia Research Consortium annual meeting. Additionally, we plan to publish findings from this study in peer-reviewed scientific journals, international conferences such as the AAIC, INTERSPEECH and ICASSP and communicate the study results to participants, healthcare professionals and the public by publishing a lay language report on the university website. We plan to publish a description of the data in venues like Nature Scientific Data and present it at the Language resources and evaluation conference (LREC).

DATA STORAGE

During the study, data will be securely stored on the University of Edinburgh's DataStore service, where it will be accessible only to the research team and not shared

For peer review only

For peer review only - http://bmjopen.bmj.com/site/about/guidelines.xhtml

outside of the research team in order to protect confidentiality. No personal information about potential participants will be collected as study team will not have access to the individuals' contact details who have been disseminated information about the SIDE-AD study by their parent cohort or NHS clinical teams. Personal information about enrolled participants will be stored securely and separately from research data.

After the study, for longer term preservation of the project's legacy and to ensure reproducibility of our analyses, anonymised research data will be stored on the University of Edinburgh Datashare[1], managed by the University Research Data Service, where the data will be assigned a Digital Object Identifier (DOI). As this project collects voice data, it is not possible to anonymise the data entirely.

Interested parties can contact the Primary Investigator for request for data and access can be provided on reasonable request with relevant regulatory guidance in place.

ACKNOWLEDGEMENTS

Thank you to the participants enrolled in the SIDE-AD study. We acknowledge Sony Research Awards for their funding of the project and thank the Sony team for their contribution to our thinking around this work.

AUTHOR CONTRIBUTIONS:

SS created a first draft of the protocol with input from SL and FH. SL conceived the idea and design for the study. FH developed the survey software and data management and analysis' plan with input from SS and SL. SS completed the regulatory approvals with input from FH and SL. FH and SS also performed patient and public involvement. SS revised the protocol per suggestions from SL, CWR, GMT. All authors read and approved the final protocol and manuscript for the current paper.

FUNDING STATEMENT: This work was supported by Sony Research Award grant number 11477634- c-00008809.

COMPETING INTERESTS STATEMENT.

Authors declare no competing interests. The funder has no role in the study design; collection, management, analysis or interpretation of data; and in the writing of the report. Because the funder can request that confidential information that belongs to the funder does not get disclosed and that any patentable inventions are protected, the funder is involved in the decision to submit the report for publication. However, if these conditions are met, then the authors have the ultimate authority over the decision to submit the report for publication.

[1] https://datashare.ed.ac.uk/handle/10283/8553

For peer review only - http://bmjopen.bmj.com/site/about/guidelines.xhtml

For peer review only

REFERENCES

Abdalla M, Rudzicz F, Hirst G. Rhetorical structure and Alzheimer’s disease. Aphasiology. 2018;32(1):41-60.

Ahmad FB, Anderson RN. The Leading Causes of Death in the US for 2020. Jama. 2021;325(18):1829-30.

Babulal GM, Ghoshal N, Head D, Vernon EK, Holtzman DM, Benzinger TL, Fagan AM, Morris JC, Roe CM. Mood changes in cognitively normal older adults are linked to Alzheimer disease biomarker levels. The American journal of geriatric psychiatry. 2016 Nov 1;24(11):1095-104.

Ballardsych C, Gauthier S, Corbett A, Brayne C, Aarsland D, Jones E. Alzheimer's disease. Lancet (London, England). 2011;377(9770):1019-31.

Budd Haeberlein, S., Aisen, P. S., Barkhof, F., Chalkias, S., Chen, T., Cohen, S., Dent, G., Hansson, O., Harrison, K., von Hehn, C., Iwatsubo, T., Mallinckrodt, C., Mummery, C. J., Muralidharan, K. K., Nestorov, I., Nisenbaum, L., Rajagovindan, R., Skordos, L., Tian, Y., van Dyck, C. H., … Sandrock, A. (2022). Two Randomized Phase 3 Studies of Aducanumab in Early Alzheimer's Disease. The journal of prevention of Alzheimer's disease, 9(2), 197–210. https://doi.org/10.14283/jpad.2022.30

Chan A-W, Tetzlaff JM, Gøtzsche PC, Altman DG, Mann H, Berlin J, Dickersin K, Hróbjartsson A, Schulz KF, Parulekar WR, Krleža-Jerić K, Laupacis A, Moher D. SPIRIT 2013 Explanation and Elaboration: Guidance for protocols of clinical trials. BMJ. 2013;346:e7586

De la Fuente Garcia S, Ritchie CW, Luz S. Artificial Intelligence, Speech, and Language Processing Approaches to Monitoring Alzheimer’s Disease: A Systematic Review. Journal of Alzheimer's Disease. 2020;78:1547-74.

DeTure MA, Dickson DW. The neuropathological diagnosis of Alzheimer’s disease. Molecular Neurodegeneration. 2019;14(1):32.

De la Fuente Garcia S, Haider F, Luz S. COVID-19: Affect recognition through voice analysis during the winter lockdown in Scotland. In2021 43rd Annual International Conference of the IEEE Engineering in Medicine & Biology Society (EMBC) 2021 Nov 1 (pp. 2326-2329). IEEE.

For peer review only - http://bmjopen.bmj.com/site/about/guidelines.xhtml

de Lope, J., Graña, M. 2023. An ongoing review of speech emotion recognition. Neurocomputing. 528 (1-11) https://doi.org/10.1016/j.neucom.2023.01.002

Eyben F, Scherer KR, Schuller BW, et al. The Geneva Minimalistic Acoustic Parameter Set (GeMAPS) for voice research and affective computing. IEEE Trans Affect Comput 2016;7:190–202.

Eyigoz E, Mathur S, Santamaria M, Cecchi G, Naylor M. Linguistic markers predict onset of Alzheimer's disease. EClinicalMedicine. 2020;28:100583. Published 2020 Oct 22. doi:10.1016/j.eclinm.2020.100583

Gauthier S, Rosa-Neto, P., Morais, J.A., Webster, C. Alzheimer's Disease International. World Alzheimer Report. Journey through the diagnosis of dementia. 2021

Golde TE. Alzheimer's disease – the journey of a healthy brain into organ failure. Molecular Neurodegeneration. 2022;17(1):18.

Haider, F., De La Fuente, S. and Luz, S., 2019. An assessment of paralinguistic acoustic features for detection of Alzheimer's dementia in spontaneous speech. IEEE Journal of Selected Topics in Signal Processing, 14(2), pp.272-281.

Landes AM, Sperry SD, Strauss ME. Prevalence of apathy, dysphoria, and depression in relation to dementia severity in Alzheimer's disease. The Journal of neuropsychiatry and clinical neurosciences. 2005 Aug;17(3):342-9.

Lim YY, Maruff P, Pietrzak RH, Ames D, Ellis KA, Harrington K, et al. Effect of amyloid on memory and non-memory decline from preclinical to clinical Alzheimer's disease. Brain. 2014;137(Pt 1):221-31.

Luz S. Longitudinal Monitoring and Detection of Alzheimer's Type Dementia from Spontaneous Speech Data. In Computer Based Medical Systems. Institute of Electrical and Electronics Engineers (IEEE). 2017. p. 45-46.

Luz S, Haider F, Fromm D, MacWhinney B, (eds.). Alzheimer's Dementia Recognition Through Spontaneous Speech. Lausanne, Switzerland: Frontiers Media S.A., 2021. 258 p.

For peer review only - http://bmjopen.bmj.com/site/about/guidelines.xhtml

For peer review only

Martínez-Nicolás, I., Llorente, T. E., Martínez-Sánchez, F., & Meilán, J. J. G. (2021). Ten Years of Research on Automatic Voice and Speech Analysis of People With Alzheimer's Disease and Mild Cognitive Impairment: A Systematic Review Article. Frontiers in psychology, 12, 620251. https://doi.org/10.3389/fpsyg.2021.620251

McDade E, Wang G, Gordon BA, Hassenstab J, Benzinger TLS, Buckles V, et al. Longitudinal cognitive and biomarker changes in dominantly inherited Alzheimer disease. Neurology. 2018;91(14):e1295-e306.

Posner H, Curiel R, Edgar C, Hendrix S, Liu E, Loewenstein DA, et al. Outcomes Assessment in Clinical Trials of Alzheimer's Disease and its Precursors: Readying for Short-term and Long-term Clinical Trial Needs. Innovations in clinical neuroscience. 2017;14(1-2):22-9.

Rashad A, Rasool A, Shaheryar M, et al. Donanemab for Alzheimer's Disease: A Systematic Review of Clinical Trials. Healthcare (Basel). 2022;11(1):32. Published 2022 Dec 22. doi:10.3390/healthcare11010032

Raudys SJ, Jain AK. Small sample size effects in statistical pattern recognition: recommendations for practitioners. IEEE Trans PatternAnal Mach Intell 1991;13:252–64.

Riley RD, Ensor J, Snell KI, Harrell FE, Martin GP, Reitsma JB, Moons KG, Collins G, Van Smeden M. Calculating the sample size required for developing a clinical prediction model. Bmj. 2020 Mar 18;368.

Ritchie, C. W., & Ritchie, K. (2012). The PREVENT study: a prospective cohort study to identify mid-life biomarkers of late-onset Alzheimer's disease. BMJ open, 2(6), e001893. https://doi.org/10.1136/bmjopen-2012-001893

Silva MVF, Loures CdMG, Alves LCV, de Souza LC, Borges KBG, Carvalho MdG. Alzheimer's disease: risk factors and potentially protective measures. Journal of Biomedical Science. 2019;26(1):33.

Vrahatis, Aristidis G., Konstantina Skolariki, Marios G. Krokidis, Konstantinos Lazaros, Themis P. Exarchos, and Panagiotis Vlamos. 2023. "Revolutionizing the Early Detection

For peer review only - http://bmjopen.bmj.com/site/about/guidelines.xhtml

of Alzheimer's Disease through Non-Invasive Biomarkers: The Role of Artificial Intelligence and Deep Learning" Sensors 23, no. 9: 4184. https://doi.org/10.3390/s23094184

Williams, E., McAuliffe, M., & Theys, C. (2021). Language changes in Alzheimer's disease: A systematic review of verb processing. Brain and language, 223, 105041. https://doi.org/10.1016/j.bandl.2021.105041

Öhman F, Hassenstab J, Berron D, Schöll M, Papp KV. Current advances in digital cognitive assessment for preclinical Alzheimer's disease. Alzheimer's & dementia (Amsterdam, Netherlands). 2021;13(1):e12217-e.

For peer review only

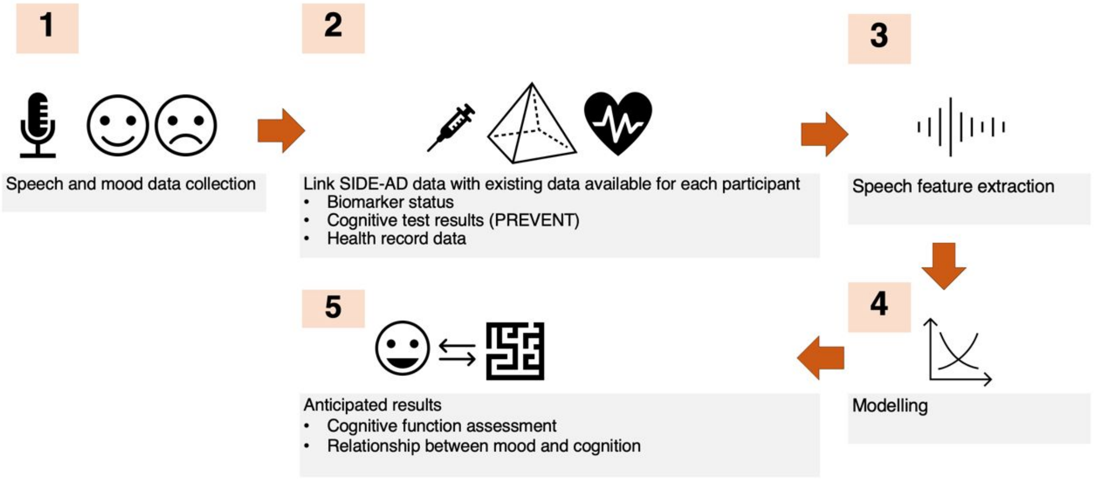

For peer review only

Participant Information Sheet

Speech for Intelligent cognition change tracking and DEtection of Alzheimer's Disease Research Program: SIDE-AD

Chief Investigator:
Saturnino Luz, PhD

Research team address:
University of Edinburgh
Usher Institute of Public Health
9 Little France Road
Edinburgh BioQuarter
EH16 4UX
side-ad@ed.ac.uk

You are being invited to take part in a research study. Before you decide whether or not to take part, please take time to read the following information carefully. Talk to your family about the study if you wish.

For peer review only

What is the purpose of the study?

Each year we understand more about brain health and how this relates to dementia. Although the brain changes for all of us as we age, and many changes are normal, abnormal changes can be due to an underlying brain disease. One of the most common brain diseases is Alzheimer's disease, which causes dementia.

We now know that when Alzheimer's disease develops, there are changes in the brain which may occur many years before memory and thinking skills are affected. This is typically many years before somebody is ultimately diagnosed with dementia. Throughout this information sheet, when we talk about Alzheimer's disease, we mean the underlying brain disease that over many years may lead to Alzheimer's dementia. Currently, doctors can only diagnose Alzheimer's disease at the most advanced stage when people already have dementia or are very close to developing it.

The purpose of this study is to understand more about speech as potential early indicator of dementia risk. This is because changes in how somebody speaks may show markers of pathological changes in Alzheimer's disease. The SIDE-AD study is a

For peer review only - http://bmjopen.bmj.com/site/about/guidelines.xhtml

longitudinal cohort study which means that if you take part in the study, you will be emailed a follow-up email to carry out follow-up assessments. Taking part in the study and in the follow-up assessments is all voluntary.

This study aims to develop digital speech-based markers for Alzheimer's disease. Please note that the speech assessment is not a diagnostic tool for Alzheimer's disease but is used to study the disease.

Why have I been invited to take part?

You have been approached because you are either healthy and do not experience cognitive decline or you are engaged with secondary services for cognitive decline and may or may not have a diagnosis related to memory concerns.

We invite individuals through the following paths:

- Taking part in the PREVENT cohort study
- Attending NHS memory assessment services

We aim to recruit 350 participants from the PREVENT cohort study and 150 participants from NHS services.

To find out which factors may predict risk of Alzheimer's dementia, we need a broad range of people with different likelihoods of developing Alzheimer's dementia. We do not know in advance who will develop dementia and who will not. This means we aim to include in the SIDE-AD study people who are possibly at high risk, low risk and anywhere in between. As we follow all the people in the SIDE-AD study, we learn which factors predict risk of dementia. Only by including a wide range of people can we identify risk factors Alzheimer's dementia.

Research team address:
University of Edinburgh
Usher Institute of Public Health
9 Little France Road
Edinburgh BioQuarter
EH16 4UX; side-ad@ed.ac.uk

For peer review only - http://bmjopen.bmj.com/site/about/guidelines.xhtml

THE UNIVERSITY of EDINBURGH

NHS Lothian

Do I have to take part?

No, it is up to you to decide whether or not to take part. If you decide to take part, you are still free to withdraw at any time and without giving a reason. Deciding not to take part or withdrawing from the study will not affect the clinical care that you receive.

What will happen if I take part?

This is a longitudinal study. Longitudinal simply means that we will send out emails for follow-up assessments every three months over two years. All assessments are done remotely at home, and you are free to withdraw at any point. Each study assessment will take about 5 min and you are asked to use either your computer or any smart device. A study assessment includes:

- *Background information about you*
- *Four questions about your mood, anxiety and apathy levels*
- *Recording yourself speak for about a minute for speech analysis*
  You could talk about any aspects about brain health. For example, you could talk about what brain health means to you, what you do to look after your brain health or what you feel you could do differently.

If you decide to take part in the study, please go to the below website:

tiny.cc/speechstudy

Before you start the study, we ask that you read this information sheet and consent to taking part in the study. Please read the statements carefully and write your initials in the box if you agree. If you have any questions about the study, please contact the research team. After you have given electronic consent to taking part in the study, the assessments will start.

Research team address:
University of Edinburgh
Usher Institute of Public Health
9 Little France Road
Edinburgh BioQuarter
EH16 4UX; side-ad@ed.ac.uk

For peer review only - http://bmjopen.bmj.com/site/about/guidelines.xhtml

THE UNIVERSITY of EDINBURGH

NHS Lothian

Please note that on the consent form, we will also ask for your permission to be contacted to discuss record linkage. If you consent to this, a member of our research team will contact you and have an informed discussion with you whether you agree to record linkage and take your consent. Record linkage is applicable for participants who are recruited through clinical services and means that we can match your study data to your health records collected during your routine NHS care. We will do this using your name and date of birth. This can be really useful to researchers to understand what research data means in relation to clinical findings such as brain scans, routine blood tests and treatment decisions or diagnosis. You do not have to agree to this to be in the study.

if you are having problems completing the study activities during the course of the study (meaning you have difficulties understanding information and making a decision based on it), you can still remain in the study unless you decide to leave. This simply means that whether you experience problems with your brain health such as remembering things and planning or you do not experience these kinds of problems, you are welcome to continue taking part in our study. if you are having problems completing the study activities, you are welcome to get help from friends or family with any aspects of the study.

At any stage in the study, if you no longer wish to continue, you can withdraw from either all aspects of the study but allow us to use data collected up to the point where you discontinue **OR** you can withdraw from all aspects of the study with removal of all previously collected data. You simply have to tell us what you prefer. It is important to realise that when you start the study you are free to leave at any time point after giving consent.

Research team address:
University of Edinburgh
Usher Institute of Public Health
9 Little France Road
Edinburgh BioQuarter
EH16 4UX; side-ad@ed.ac.uk

For peer review only - http://bmjopen.bmj.com/site/about/guidelines.xhtml

THE UNIVERSITY of EDINBURGH

NHS Lothian

What are the possible benefits of taking part?

You should not expect to benefit medically from being involved in this project. Participants can find participating in studies like this enjoyable.

Your contribution to this research enables us to further our understanding of Alzheimer's disease. Specifically, taking part in the study may help researchers and clinicians in identifying early signs of Alzheimer's disease and ultimately to treat or prevent dementia.

What are the possible disadvantages of taking part?

We appreciate that taking part in the study assessments is a time commitment and we will do everything we can to fit into your schedule and be as flexible as possible.

The study is entirely remote which means you can choose the time when is suitable for you to do the study assessments.

What if there are any problems?

If you have a concern about any aspect of this study, please contact the study team who will be delighted to answer any questions about the study. If you would like to discuss this study with someone independent of the study, please contact Dr Miles Welstead (Miles.Welstead@ed.ac.uk) who will do his best to answer your questions. However, if you are still unhappy with the answers given and wish to complain, you can do this through the NHS Lothian Patient Experience Team (2nd Floor; Waverley Gate; 2 – 4 Waterloo Place; Edinburgh; EH1 3EG; Tel: 0131 536 3370; email: feedback@nhslothian.scot.nhs.uk).

Will my taking part be kept confidential?

You will be assigned a participant number and you will not be personally identified in any of the research data. Your personal data, including your voice recordings, will not leave the research site. Your voice data will be stored on secure

Research team address:
University of Edinburgh
Usher Institute of Public Health
9 Little France Road
Edinburgh BioQuarter
EH16 4UX; side-ad@ed.ac.uk

For peer review only

For peer review only - http://bmjopen.bmj.com/site/about/guidelines.xhtml

THE UNIVERSITY of EDINBURGH

NHS Lothian

University of Edinburgh servers (servers are large storage spaces for computer data and be password protected) and will only be accessible to legitimate members of the research team and is approved by the sponsor. In order to monitor and audit the study we will ask for your consent for responsible representatives from the study sponsor and NHS Institution(s) to access your data collected during the study. The Sponsors are responsible for overall management of the study and providing insurance and indemnity. The sponsor is the University of Edinburgh and NHS Lothian under a joint agreement between those two organisations called ACCORD.

What will happen to the results of the study?

To analyse early indicators of Alzheimer's disease, we pool together data from all the study participants. This means that we do not analyse individual participant trajectories (changes in risk factors over time) but look for patterns across all the study participants.

External researchers will be able to apply to access research data from the SIDE-AD study. Please note that your voice recordings will not be shared. Research data is anonymous, and you will not be identifiable in any of this data. We think this is important to maximise the use of all data collected in the study. Sony Research Awards who funds this study may have access to the anonymised results of this study.

Your voice data may be transcribed by an approved 3rd party vendor. This means that the transcribing company we use will respect the confidentiality of your data and their work has been approved by the University of Edinburgh. Transcribing means writing out all the spoken information you give and we record into text. All data transcripts will be anonymised. We will prepare scientific publications based on the findings from the SIDE-AD study which will be presented at national and international conferences. You will not be identified in any of the publications. We will communicate findings from these studies in regular newsletters, which you can sign up to receive.

Research team address:
University of Edinburgh
Usher Institute of Public Health
9 Little France Road
Edinburgh BioQuarter
EH16 4UX; side-ad@ed.ac.uk

For peer review only

For peer review only - http://bmjopen.bmj.com/site/about/guidelines.xhtml

Where can you find out more about how your information is used?

You can find out more about how we use your information

- at www.hra.nhs.uk/information-about-patients/
- our leaflet available from www.hra.nhs.uk/patientdataandresearch
- by asking one of the research team
- by sending an email to the Sponsor's Data Protection Officer dpo@ed.ac.uk

Who is organising and funding the research?

This study is led by the University of Edinburgh, Dr Saturnino Luz. The other team members include Dr Fasih Haider and Dr Stina Saunders who are both postdoctoral research fellows at the University of Edinburgh. This study is funded by Sony Research Awards.

Who has reviewed the study?

All research in the NHS is looked at by an independent group of people called a Research Ethics Committee. A favourable ethical opinion has been obtained from XXXX. NHS management approval has also been given. IRAS Project ID 323311.

Researcher Contact Details

For any questions concerns, please email Dr Stina Saunders (Stina.Saunders@ed.ac.uk) or dedicated study contact email side-ad@ed.ac.uk.
University of Edinburgh
Usher Institute of Public Health
9 Little France Road
Edinburgh BioQuarter
Edinburgh
EH16 4UX

Independent Contact Details

If you would like to discuss this study with someone independent of the study, please contact Dr Miles Welstead (Miles.Welstead@ed.ac.uk)

Complaints

If you wish to make a complaint about the study please contact:
Patient Experience Team
2 – 4 Waterloo Place, Edinburgh, EH1 3EG
feedback@nhslothian.scot.nhs.uk
0131 536 3370

Thank you for considering your involvement in this project and taking time to read this information sheet

For peer review only - http://bmjopen.bmj.com/site/about/guidelines.xhtml

This document is for review purposes only, consent takes place electronically using the online study platform

Electronic Informed Consent Form for Participants

Speech for Intelligent cognition change tracking and DEtection of AD (SIDE-AD) Research Program: SIDE-AD

1. I confirm that I have read and understand the information sheet (SIDE-AD PIS v3 20 Sep 2023) for the above study. I have had the opportunity to consider the information, ask questions and have had these questions answered satisfactorily.
2. I understand that my participation is voluntary and that I am free to withdraw at any time without giving any reason and without my medical care and/or legal rights being affected.
3. I understand that data collected about me during the study may be converted to anonymised data.
4. I understand that relevant sections of my data collected during the study may be looked at by individuals from the Sponsor (University of Edinburgh and NHS Lothian under a joint agreement between those two organisations called ACCORD) and/or from regulatory authorities where it is relevant to my taking part in this research. I give permission for these individuals to have access to my data and/or medical records.
5. I agree to my voice being recorded as part of the study.
6. I understand that if I lose capacity during the course of this study, I will remain in the current study or I am free to withdraw. This means that whether I experience problems with my brain health or I do not experience problems, I am welcome to continue taking part in this study.
7. I agree to my audio recordings being transcribed by a third-party contractor.
8. I understand that information collected will be managed by the research team only and will be destroyed after a period of ten years and any information collected will be kept in line with the data protection act in a secure way.
9. I agree to my anonymised data being used in future studies.
10. I agree to be re-contacted about future research.
11. I confirm that I am over 40 years of age.
12. I agree to take part in the above study.
Final statement is only applicable to individuals recruited through NHS memory clinics.<br><br>***Please leave this blank if you are a PREVENT study research participant***.<br><br>*If you agree to this statement, a member of the research team will contact you to explain this further and ask for your consent to access relevant sections of your Medical Records.*

For peer review only - http://bmjopen.bmj.com/site/about/guidelines.xhtml

*Agreeing to this statement **does not grant access** to your Medical Records. Please enter **initials** below if you agree.*
**13. Optional:** I agree to be contacted about the potential to link my study data with my NHS Medical Records.

For peer review only

Reporting checklist for protocol of a clinical trial.

Based on the SPIRIT guidelines.

Instructions to authors

Complete this checklist by entering the page numbers from your manuscript where readers will find each of the items listed below.

Your article may not currently address all the items on the checklist. Please modify your text to include the missing information. If you are certain that an item does not apply, please write "n/a" and provide a short explanation.

Upload your completed checklist as an extra file when you submit to a journal.

In your methods section, say that you used the SPIRITreporting guidelines, and cite them as:

Chan A-W, Tetzlaff JM, Gøtzsche PC, Altman DG, Mann H, Berlin J, Dickersin K, Hróbjartsson A, Schulz KF, Parulekar WR, Krleža-Jerić K, Laupacis A, Moher D. SPIRIT 2013 Explanation and Elaboration: Guidance for protocols of clinical trials. BMJ. 2013;346:e7586

		Reporting Item	Page Number
**Administrative information**			
Title	#1	Descriptive title identifying the study design, population, interventions, and, if applicable, trial acronym	1
Trial registration	#2a	Trial identifier and registry name. If not yet registered, name of intended registry	n/a
Trial registration: data set	#2b	All items from the World Health Organization Trial Registration Data Set	n/a
Protocol version	#3	Date and version identifier	5
Funding	#4	Sources and types of financial, material, and other support	13
Roles and responsibilities: contributorship	#5a	Names, affiliations, and roles of protocol contributors	13 and title page

For peer review only

Roles and responsibilities: sponsor contact information	#5b	Name and contact information for the trial sponsor	11
Roles and responsibilities: sponsor and funder	#5c	Role of study sponsor and funders, if any, in study design; collection, management, analysis, and interpretation of data; writing of the report; and the decision to submit the report for publication, including whether they will have ultimate authority over any of these activities	13
Roles and responsibilities: committees	#5d	Composition, roles, and responsibilities of the coordinating centre, steering committee, endpoint adjudication committee, data management team, and other individuals or groups overseeing the trial, if applicable (see Item 21a for data monitoring committee)	n/a
**Introduction**			
Background and rationale	#6a	Description of research question and justification for undertaking the trial, including summary of relevant studies (published and unpublished) examining benefits and harms for each intervention	3
Background and rationale: choice of comparators	#6b	Explanation for choice of comparators	n/a
Objectives	#7	Specific objectives or hypotheses	5
Trial design	#8	Description of trial design including type of trial (eg, parallel group, crossover, factorial, single group), allocation ratio, and framework (eg, superiority, equivalence, non-inferiority, exploratory)	5
**Methods: Participants, interventions, and outcomes**			
Study setting	#9	Description of study settings (eg, community clinic, academic hospital) and list of countries where data will be collected. Reference to where list of study sites can be obtained	5
Eligibility criteria	#10	Inclusion and exclusion criteria for participants. If applicable, eligibility criteria for study centres and individuals who will	6

For peer review only

		perform the interventions (eg, surgeons, psychotherapists)	
Interventions: description	#11a	Interventions for each group with sufficient detail to allow replication, including how and when they will be administered	n/a
Interventions: modifications	#11b	Criteria for discontinuing or modifying allocated interventions for a given trial participant (eg, drug dose change in response to harms, participant request, or improving / worsening disease)	n/a
Interventions: adherance	#11c	Strategies to improve adherence to intervention protocols, and any procedures for monitoring adherence (eg, drug tablet return; laboratory tests)	n/a
Interventions: concomitant care	#11d	Relevant concomitant care and interventions that are permitted or prohibited during the trial	n/a
Outcomes	#12	Primary, secondary, and other outcomes, including the specific measurement variable (eg, systolic blood pressure), analysis metric (eg, change from baseline, final value, time to event), method of aggregation (eg, median, proportion), and time point for each outcome. Explanation of the clinical relevance of chosen efficacy and harm outcomes is strongly recommended	7
Participant timeline	#13	Time schedule of enrolment, interventions (including any run-ins and washouts), assessments, and visits for participants. A schematic diagram is highly recommended (see Figure)	5 and 9
Sample size	#14	Estimated number of participants needed to achieve study objectives and how it was determined, including clinical and statistical assumptions supporting any sample size calculations	6
Recruitment	#15	Strategies for achieving adequate participant enrolment to reach target sample size	5
**Methods: Assignment of interventions (for controlled trials)**			
Allocation: sequence generation	#16a	Method of generating the allocation sequence (eg, computer-generated random numbers), and list of any factors for stratification. To reduce predictability of a random sequence, details of any planned restriction (eg, blocking) should be provided in a separate document that is unavailable to those who enrol participants or assign interventions	n/a

For peer review only

Allocation concealment mechanism	#16b	Mechanism of implementing the allocation sequence (eg, central telephone; sequentially numbered, opaque, sealed envelopes), describing any steps to conceal the sequence until interventions are assigned	n/a
Allocation: implementation	#16c	Who will generate the allocation sequence, who will enrol participants, and who will assign participants to interventions	n/a
Blinding (masking)	#17a	Who will be blinded after assignment to interventions (eg, trial participants, care providers, outcome assessors, data analysts), and how	n/a
Blinding (masking): emergency unblinding	#17b	If blinded, circumstances under which unblinding is permissible, and procedure for revealing a participant's allocated intervention during the trial	n/a
**Methods: Data collection, management, and analysis**			
Data collection plan	#18a	Plans for assessment and collection of outcome, baseline, and other trial data, including any related processes to promote data quality (eg, duplicate measurements, training of assessors) and a description of study instruments (eg, questionnaires, laboratory tests) along with their reliability and validity, if known. Reference to where data collection forms can be found, if not in the protocol	7
Data collection plan: retention	#18b	Plans to promote participant retention and complete follow-up, including list of any outcome data to be collected for participants who discontinue or deviate from intervention protocols	10
Data management	#19	Plans for data entry, coding, security, and storage, including any related processes to promote data quality (eg, double data entry; range checks for data values). Reference to where details of data management procedures can be found, if not in the protocol	12
Statistics: outcomes	#20a	Statistical methods for analysing primary and secondary outcomes. Reference to where other details of the statistical analysis plan can be found, if not in the protocol	10
Statistics: additional analyses	#20b	Methods for any additional analyses (eg, subgroup and adjusted analyses)	10

For peer review only

Statistics: analysis population and missing data	#20c	Definition of analysis population relating to protocol non-adherence (eg, as randomised analysis), and any statistical methods to handle missing data (eg, multiple imputation)	10
**Methods: Monitoring**			
Data monitoring: formal committee	#21a	Composition of data monitoring committee (DMC); summary of its role and reporting structure; statement of whether it is independent from the sponsor and competing interests; and reference to where further details about its charter can be found, if not in the protocol. Alternatively, an explanation of why a DMC is not needed	n/a
Data monitoring: interim analysis	#21b	Description of any interim analyses and stopping guidelines, including who will have access to these interim results and make the final decision to terminate the trial	10
Harms	#22	Plans for collecting, assessing, reporting, and managing solicited and spontaneously reported adverse events and other unintended effects of trial interventions or trial conduct	n/a
Auditing	#23	Frequency and procedures for auditing trial conduct, if any, and whether the process will be independent from investigators and the sponsor	n/a
**Ethics and dissemination**			
Research ethics approval	#24	Plans for seeking research ethics committee / institutional review board (REC / IRB) approval	2
Protocol amendments	#25	Plans for communicating important protocol modifications (eg, changes to eligibility criteria, outcomes, analyses) to relevant parties (eg, investigators, REC / IRBs, trial participants, trial registries, journals, regulators)	6
Consent or assent	#26a	Who will obtain informed consent or assent from potential trial participants or authorised surrogates, and how (see Item 32)	8
Consent or assent: ancillary studies	#26b	Additional consent provisions for collection and use of participant data and biological specimens in ancillary studies, if applicable	n/a
Confidentiality	#27	How personal information about potential and enrolled participants will be collected, shared, and maintained in order to protect	12

For peer review only

		confidentiality before, during, and after the trial	
Declaration of interests	#28	Financial and other competing interests for principal investigators for the overall trial and each study site	13
Data access	#29	Statement of who will have access to the final trial dataset, and disclosure of contractual agreements that limit such access for investigators	13
Ancillary and post trial care	#30	Provisions, if any, for ancillary and post-trial care, and for compensation to those who suffer harm from trial participation	n/a
Dissemination policy: trial results	#31a	Plans for investigators and sponsor to communicate trial results to participants, healthcare professionals, the public, and other relevant groups (eg, via publication, reporting in results databases, or other data sharing arrangements), including any publication restrictions	12
Dissemination policy: authorship	#31b	Authorship eligibility guidelines and any intended use of professional writers	n/a
Dissemination policy: reproducible research	#31c	Plans, if any, for granting public access to the full protocol, participant-level dataset, and statistical code	12
**Appendices**			
Informed consent materials	#32	Model consent form and other related documentation given to participants and authorised surrogates	1
Biological specimens	#33	Plans for collection, laboratory evaluation, and storage of biological specimens for genetic or molecular analysis in the current trial and for future use in ancillary studies, if applicable	n/a

None The SPIRIT Explanation and Elaboration paper is distributed under the terms of the Creative Commons Attribution License CC-BY-NC. This checklist can be completed online using https://www.goodreports.org/, a tool made by the EQUATOR Network in collaboration with Penelope.ai

For peer review only

BMJ Open

Protocol: Longitudinal observational cohort study: Speech for Intelligent cognition change tracking and DEtection of Alzheimer's Disease (SIDE-AD)

Journal:	*BMJ Open*
Manuscript ID	bmjopen-2023-082388.R1
Article Type:	Protocol
Date Submitted by the Author:	09-Feb-2024

Complete List of Authors:	Saunders, Stina; University of Edinburgh<br>Haider, Fasih; University of Edinburgh, Usher Institute<br>Ritchie, Craig; Centre for Clinical Brain Sciences, Department of Psychiatry, University of Edinburgh; Scottish Brain Sciences<br>Muniz Terrera, Graciela; University of Edinburgh, Centre for Dementia Prevention; Ohio University, Heritage College of Osteopathic Medicine<br>Luz, Saturnino; University of Edinburgh School of Molecular Genetic and Population Health Sciences, Usher Institute of Population Health Sciences and Informatics

**Primary Subject Heading**:	Neurology
Secondary Subject Heading:	Global health
Keywords:	NEUROLOGY, PUBLIC HEALTH, Clinical Trial

SCHOLARONE™
Manuscripts

Protocol: Longitudinal observational cohort study: Speech for Intelligent cognition change tracking and DEtection of Alzheimer's Disease (SIDE-AD)

Saunders, S.[1] (PhD), Haider, F. (PhD)[1], Ritchie, C.W.[2,3] (MD PhD), Muniz-Terrera, G.[3,4] (PhD), Luz, S.[1] (PhD)

[1]Usher Institute; Edinburgh Medical School, University of Edinburgh, Edinburgh, UK

[2] Scottish Brain Sciences, Edinburgh, UK

[3]Centre for Clinical Brain Sciences, University of Edinburgh, Edinburgh, UK

[4]Ohio University, Heritage College of Osteopathic Medicine, Ohio, US

Corresponding author: Stina Saunders (Stina.Saunders@ed.ac.uk)

Stina Saunders
Usher Institute
University of Edinburgh

Declarations of interest: none (all authors)

Word count 4,204

For peer review only

ABSTRACT

Introduction

There is emerging evidence that speech may be a potential indicator and manifestation of early Alzheimer's disease (AD) pathology. Therefore, the University of Edinburgh and Sony Research have partnered to create the SIDE-AD (Speech for Intelligent cognition change tracking and DEtection of Alzheimer's Disease) study which aims to develop digital speech-based biomarkers for use in neurodegenerative disease.

Methods and Analysis

SIDE-AD is an observational longitudinal study, collecting samples of spontaneous speech. Participants are recruited from existing cohort studies as well as NHS memory clinics in Scotland. Using an online platform, participants record a voice sample talking about their brain health and rate their mood, anxiety and apathy. The speech biomarkers will be analysed longitudinally, and we will use machine learning and natural language processing technology to automate the assessment of the respondents' speech patterns.

Ethics and Dissemination

The SIDE-AD study has been approved by the NHS Research Ethics Committee (REC reference: 23/WM/0153, protocol number AC23046, IRAS Project ID 323311) and received NHS management approvals from Lothian, Fife and Forth Valley NHS boards. Our main ethical considerations pertain to the remote administration of the study, such as taking remote consent. To address this, we implemented a consent process whereby the first step of the consent is done entirely remotely but a member of the research team contacts the participant over the phone to consent participants to the most sensitive, optional, elements of the study. Results will be presented at conferences, published in peer reviewed journals, and communicated to study participants.

Keywords: speech analysis; Alzheimer's disease; early detection

For peer review only

ARTICLE SUMMARY

Strengths and limitations of this study

- The main strength of this study is that it combines assessment of cognition with mood, anxiety and apathy assessment over time, through spontaneous speech which can be collected remotely, unobtrusively and at scale.
- Another strength is its ability to relate patterns of speech collected while the participant is talking about their brain health to cognitive, clinical and functional outcomes through existing cohort data, and NHS patient record data (optional consent).
- Assessment by analysis of spontaneous speech, by relying on an inherently repeatable task that is not prone to practice effects, provides a way of avoiding variability in cognitive assessment due to extraneous environmental or subjective factors which could distort the true trajectory of cognitive performance over time.
- A limitation of the study is that there is no way to control for variability in audio recording equipment, such as background noise, as participants will be using their own personal computers or mobile phones to collect the speech samples.
- Another possible limitation is that although the study will collect a standardised self-assessment baseline, there may be heterogeneity of data between the recruitment sources, which complicates the analysis.

INTRODUCTION

In the current study, we will be analysing short recordings of individuals' speech to detect early changes indicative of cognitive decline in the older adult population. The following section contextualises the emergence of speech-based "digital biomarkers" in the neurodegenerative disease area.

Dementia is a major public health concern with over 55 million people currently living with dementia worldwide and recent projections reaching 78 million living with dementia by 2030 (1). Alzheimer's disease (AD) is the most common underlying pathology for the dementia syndrome, accounting for 60 to 70% of all dementia cases (2; 3) and is also one of the leading causes of death in the US (4). Due to a long preclinical period in the AD pathology, dementia is a late-stage manifestation of illness that has been developing years before dementia manifests (5; 6). Though not yet globally available, recent pharmacological advances have yielded several new medications for the treatment of AD (e.g., 7; 8) and there are many other pharmacological approaches in progress which necessitate detection of AD in its earliest pathological stage.

Dementia is an illness that causes neurodegeneration whereby the brain gradually transitions from a healthy brain to brain failure (9). In the initial symptomatic stages, deterioration of brain health manifests as cognitive impairment, though over time once dementia develops, the cognitive impairment cascades into problems with daily functioning. Additionally, symptomatic AD may change an individual's language,

For peer review only - http://bmjopen.bmj.com/site/about/guidelines.xhtml

especially certain elements in speech such as elaboration (e.g., making an argument clearer or providing examples) and attribution (e.g., the ability to attribute characteristics to others) (10). What is more, evidence suggests that there is measurable difference between the speech patterns for individuals who are ageing with and without cognitive decline. For example, AD has been associated with shorter word lengths, higher pronoun-to-noun ratio, and reduced use of nouns (11); reduced meaningful content expressed, a reduction of total output of expressive language and a preference for generic verbs and simple syntax (12). Acoustic features related to prosody and speech rhythms have also been found to be good predictors of AD (13; 14).

The significance of speech-based markers relates both to research and clinical practice. For instance, though the primary outcomes measured in AD clinical trials focus on cognition and biomarker data such as brain imaging, analysing cerebrospinal fluid, or blood tests (15), speech analysis could become a promising tool for detecting early neurodegenerative disease (16). A systematic review of studies looking to distinguish healthy ageing from cognitive decline in older adults using speech features concluded that most studies record a diagnostic accuracy over 88% for AD and 80% for MCI (13). Furthermore, a study by Eyigoz et al. (2020) (17) predicted future diagnosis of AD among a cognitively normal baseline of Framingham Heart Study participants and demonstrated that linguistic features from a single administration of the cookie-theft picture description task out-performed predictive models that incorporated genetic risk status, demographic variables, and neuropsychological test results. Though limitations around specificity to underlying aetiology remain (18), the ease of remote data collection makes speech biomarkers a valuable area to explore.

In our own systematic review on the potential of using interactive AI methods to detect AD biomarkers, we concluded that when compared to traditional neuropsychological assessment methods, speech and language technology were at least equally discriminative between different groups (14). Interestingly, speech-based markers have been found to detect AD pathology such as amyloid-β status (19), but evidence also suggests that people with higher amyloid levels (both asymptomatic and those with mild cognitive impairment) exhibit a greater rate of decline in episodic memory and language (20), suggesting the causal relation between speech and AD pathology should be explored further. Some of the most promising AD markers in speech may be lexico-semantic (21), meaning the use of deictic words such as those referring to specific time (e.g., now, tomorrow), place (e.g., here, at home), or person (e.g., I, the person). While research on speech biomarkers is only beginning to emerge, we expect speech and language analysis to play a role in detecting and tracking preclinical AD in the near future (22; 23).

In addition to cognition, speech and language are indicators of neurobehavioral outcomes such as mood, apathy and anxiety, and have been used as input data in machine learning based emotion recognition models (24). Automatic methods for assessment of mood, anxiety and apathy could be an important component in AD

For peer review only - http://bmjopen.bmj.com/site/about/guidelines.xhtml

detection and on-going monitoring of patient outcomes. Apathy is the most common neuropsychiatric symptom in AD patients, and its accurate detection is important in terms of treatment outcomes, and carer education and well-being (25). Furthermore, mood disorders have also been linked to AD pathology (26). However, as highlighted above, one of the limitations in speech biomarkers is that they may indicate deviations from typical function but not map to a specific neurodegenerative disease (27).

To date, most studies of neurodegenerative diseases involving speech have been laboratory-based, and rarely included spontaneous speech. However, there are advantages in collecting spontaneous connected speech. Spontaneous speech can be captured in more natural settings over time, thus mitigating problems that might affect performance in controlled, cross-sectional data, such as a participant having an "off day" or having poorly slept the night before the test. Speech can also be collected remotely, using widespread mobile devices and smartphones, which facilitates data collection at scale and over time (28). Early recognition of individuals at risk of AD is important in order to intervene at a time where any intervention may yield the most benefit.

This study explores changes in speech as potential early markers of pathological changes in AD. The SIDE-AD study is a longitudinal cohort study of individuals across the AD risk spectrum, those who are healthy volunteers and those engaged with secondary services for cognitive decline. The original contribution of our research is collecting speech data for the explicit analysis of AD speech markers, using connected speech as the most evidence-based modality for capturing relevant markers.

The study aims to develop digital speech-based biomarkers for AD in order to assess disease status in the pre-dementia stages in a real-world population.

METHODS AND ANALYSIS

This is a remote observational longitudinal study developing speech biomarkers for use in neurodegenerative disease. The SIDE-AD study involves collecting voice data using an online platform and prompting individuals to talk about brain health (Figure 1), using protocol version 4, dated 29 Nov 2023.

Figure 1. Speech for Intelligent cognition change tracking and DEtection of AD (SIDE-AD) study flow

How the sample will be selected

The selection of the participant population aims to create a risk spectrum of individuals with a low to high risk of AD. Therefore, the participant population includes individuals taking part in a longitudinal cohort study aimed at dementia prevention who are healthy volunteers but may have risk factors for dementia (such as genetic risk, increased amyloid or objective cognitive decline), individuals signed up for research registers in neurodegenerative disease and patients referred to clinical

For peer review only - http://bmjopen.bmj.com/site/about/guidelines.xhtml
For peer review only

services who present with concerns around cognitive decline in NHS memory clinics in the UK.

Participants will be identified through the different routes:

1. Participants taking part in the PREVENT Dementia study (29)

The PREVENT study is a longitudinal cohort study of individuals in mid-life with n=700 participants, creating a spectrum of individuals from low to high risk of dementia. The Participant Information Sheet (PIS) will be emailed by the PREVENT study team.

2. Participants will be identified through research registers such as Join Dementia Research (JDR), Scottish Brain Health Register (SBHR), Scottish Health Research Register (SHARE), the permission to contact list from the Scottish Dementia Research Interest Register held by the Neuroprogressive and Dementia Network (NDN)

The Participant Information Sheet (SIDE-AD study PIS) will be emailed to all eligible participants who have expressed an interest towards the SIDE-AD study.

3. Participants will be identified through NHS memory assessment services

Individuals will be either emailed the PIS electronically (Appendix 1) or handed the study PIS during routine clinic visits.

In order to achieve a spectrum of low to high individuals at risk of AD, additional recruitment sources may be added to the SIDE-AD study through protocol amendments. Protocol amendments will be communicated to relevant parties in line with regulatory requirements.

Eligibility

INCLUSION CRITERIA

- Over 40 years old
- Ability to carry out remote assessments (access to a computer/smart device)
- Have the capacity to consent
- Participating in the PREVENT study or attending an NHS memory clinic

EXCLUSION CRITERIA

- Not able to speak English
- Individuals who have not provided consent

The SIDE-AD study uses an age limit of 40 years and older to be consistent with the inclusion criteria in the PREVENT Dementia study eligibility criteria.

Sample size calculation

For peer review only

For peer review only - http://bmjopen.bmj.com/site/about/guidelines.xhtml

We aim to recruit a total of 450 participants over a 10-month period (Oct 2023-Aug 2024). The PREVENT study has 700 participants overall and at the first stage, only participants from Scotland (n=300) will be invited to take part in the SIDE-AD study. Additionally, we estimate the number of eligible individuals in NHS clinical settings to be around 150 over 10 months which will be sufficient for our planned analyses.

There is no a priori sample size calculation as the SIDE-AD study will recruit from the PREVENT study, research registers and enrich the sample with a clinical population recruiting from NHS memory clinics where all eligible individuals will be invited to take part in the SIDE-AD study. We used the Riley et al. (2020) (30) approach to calculate the sample size and elaborate on the process below.

It is difficult to estimate precisely the optimal sample sizes and measurable effect sizes for machine-learning based studies such as SIDE-AD. In the field of medical decision support systems, a commonly adopted rule of thumb for determining sample size in continuous outcome settings is to require two participants per feature (predictor) in the model. While this heuristic approach has been criticised for not taking into account the context of different studies (30), some theoretical justification for it has been offered in the pattern recognition literature for the case of classification (31). According to this approach, for relatively simple machine learning algorithms such as Euclidean distance and linear discriminant analysis, lower bounds at 1.2 and 1.4 participants per feature has been proposed, for an expected probability of misclassification (PMC) at most 50% greater than an asymptotic PMC of 0.1. Using the extended Geneva Minimalistic Acoustic Parameter Set (eGeMAPs) feature set (32) as proposed here, would therefore imply a minimum of 114 participants for a PMC at most 50% greater than a conservative asymptotic PMC of 0.1 (90% accuracy). However, this estimate presupposes the use of a fairly simple linear model. We aim to collect data from larger numbers of participants, which will allow us to experiment with larger sets of speech features. In the Riley et al. (2020) paper (3), a method is proposed for estimation of sample sizes for continuous outcomes regression models that aims to ensure that the data sample is large enough to estimate the model intercept and residual variance precisely, and account for the risk of overfitting by setting adequate regularisation and optimism targets. While this approach only covers standard regression models, it provides more conservative sample size estimates. Taking a previous study on anxiety and depression recognition from speech conducted by our group (33) as a basis, and assuming a model R2 of 0.3, a regularisation factor of 0.8, mean arousal and valence scores of 30, standard deviation of 22 and a reduced feature set of 40 features would require sample size of 421 participants, according to the formula given in Riley et al. (2020) (30). It is speculated (30) that machine learning models require much larger sample size than simpler statistical models. We intend to use existing datasets containing speech samples from over 1,000 subjects for data augmentation and dimensionality reduction methods, such as Haider et al. (2019) (34), should our study's sample sizes prove insufficient to train accurate predictive models.

For peer review only - http://bmjopen.bmj.com/site/about/guidelines.xhtml

What outcomes will be measured

As this study aims to develop models for prediction of AD and related neurobehavioural outcomes, the primary endpoints of this study are measures of model prediction performance, namely, root mean squared error (RMSE) and coefficient of determination values for predicted cognitive scores, and mood, anxiety and apathy prediction ratings, for regression models, and area under the receiver operating characteristic curve (AUROC), sensitivity and specificity for classification into score categories (e.g. high vs low mood etc).

In terms of secondary endpoints, all participants in the SIDE-AD study will self-report the following outcomes which are used as explanatory variables (Table 1).

**Variables collected as part of SIDE-AD study (all participants)**	
**Spontaneous speech**	Voice-recording in response to: "Please tell us in your own words how you look after your brain health."
**Sociodemographic details**	Date of birth<br>Gender<br>Education
**Self-reported neurodegenerative disease diagnosis**	Mild Cognitive Impairment<br>Alzheimer's disease<br>Mixed dementia<br>Dementia with Lewy bodies<br>Frontotemporal dementia<br>Vascular dementia<br>Parkinson's disease dementia<br>Parkinson's disease<br>Huntington's disease<br>Creutzfeldt-Jakob Disease<br>Korsakoff Syndrome<br>Motor neuron disease (also known as ALS or Amyotrophic lateral sclerosis)<br>Subjective Cognitive Disorder<br>Major neurocognitive disorder<br>Stroke
**Self-reported mood**	1-100 score on arousal and happiness
**Self-reported anxiety**	1-100 score
**Self-reported apathy**	1-100 score

For peer review only - http://bmjopen.bmj.com/site/about/guidelines.xhtml

Table 1 Variables collected as part of SIDE-AD study

For participants recruited through the PREVENT Dementia study, SIDE-AD will have access to the following variables collected as part of the PREVENT protocol:

- Demographics (Date of Birth; Gender; Race; Education)
- Biosamples (APOE e4 status, amyloid status)
- Risk Factors (self-reported memory concerns and health status, being out of breath, family history of dementia)
- Lifestyle Interview (Smoking status, Electronic cigarette use, Drug use, Alcohol Intake, Diet)
- Cognitive Outcomes
  - National Adult Reading Test (NARTs)
  - Visual Short Term Memory Binding Test
  - Cognito
  - ACE- III
  - Supermarket Task
  - 4 Mountains Task

- Clinical and Functional Outcomes
  - Apathy Inventory
  - Lawton Instrumental Activities Of Daily Life Scale
  - CES-D Depression Scale
  - Spielberger State Scale
  - Pittsburgh Sleep Quality Index
  - Sleep Questionnaire
  - Epworth Sleepiness Scale
  - Life Stressor Checklist
  - Brain Injury Screening Questionnaire

- Dementia Risk Score
  - CAIDE score

Individuals who are recruited through NHS services will have an optional consent for record linkage. For those participants who are recruited through NHS services and who consent to record linkage, changes in speech will be associated with routinely collected health care data.

Record linkage will include any/all the available outcomes:

- Neurodegenerative disease diagnosis
- Clinical outcomes (ADL, mood/anxiety and sleep measures)
- Neuropsychological outcomes (cognitive tests)

For peer review only - http://bmjopen.bmj.com/site/about/guidelines.xhtml

- Biomarker outcomes: Genetic risk (ApoE4)
- Biomarker outcomes: Amyloid levels
- Biomarker outcomes: Tau levels
- Neuroimaging outcomes

We note that each of the above variables will be linked with the time stamp of when this was reported (e.g., if a diagnosis is reported during one of the follow-up appointments, time since diagnosis is controlled for in the analysis).

When and how

The SIDE-AD study is self-administered remotely, though a member of the research team is available via contact details provided on the study PIS and study website. The assessment takes around 5 minutes to complete on a computer or smart device and the participant receives an email invitation for follow-up assessments every 3 months.

Interested participants follow a unique URL link to the SIDE-AD research platform, where they will be asked to give consent (Appendix 2) and perform the baseline assessment. This is done remotely from the participants' home with no direct involvement of the research team. All individuals in the SIDE-AD study will be invited to provide speech samples through the online platform. Assessments, including speech sampling, will be done quarterly over the study duration.

Participant retention and completion of follow-up is promoted through reminders in each follow-up invitation that participants' contribution is valued and voluntary.

Data analysis plan

We will conduct descriptive analysis of the sociodemographic information provided by the individuals, using simple tabulations. A lay language report with the survey results from the analysis of the data will be produced and made publicly available.

We developed the SIDE-AD platform to collect speech data and limited background/self-reported mood, anxiety and apathy data. The platform is used to monitor speech patterns over time. We will extract speech markers and evaluating a comprehensive set of acoustic features for modelling and prediction of cognitive function.

We will use the primary data for the purpose of developing models for the assessment of cognition, mood, anxiety and apathy. We will extract the following speech features and their most common statistical functionals (in-total, 88 low-level descriptors of the enhanced Geneva Minimalistic Acoustic Parameter Set [eGeMAP] as per Eyben et al., 2016) from the collected speech signal.

For peer review only - http://bmjopen.bmj.com/site/about/guidelines.xhtml

- F0 semitone: F0 is determined physiologically by the number of cycles that the vocal folds make in a second, corresponding to prosodic aspects of speech;
- Loudness: the loudness of speech implies an increase in the vocal effort which describes a strained or tense voice quality;
- Spectral flux: a low-level feature that measures how quickly the power spectrum of a signal changes, used for voice activity detection;
- Mel-frequency cepstral coefficients (MFCC):represent spectral information from the speech signal, using a scale known as the "Mel-frequency scale," which is chosen to mimic the way humans perceive audio;
- Jitter: this indicates the variability or perturbation of F0 (i.e. a measure of frequency instability)
- Shimmer: this is also also a measures of F0 perturbation but is related to the amplitude of the sound wave or the intensity of vocal emission (i.e. a measure of amplitude instability). A normal healthy voice has a small amount of instability during sustained vowel production, and tissue and muscle properties influence normal instabilities.
- F1, F2, F3: these are formant frequencies that relate to characteristics of the speaker's vocal tract;
- Alpha ratio: the ratio between the energy in the low-frequency region and the high-frequency region; a measure correlated to perceived voice quality;
- Hammarberg index: a spectral measure used as an energy distribution measure averaged across an utterance;
- Spectral Slope (linear regression within bands 0-500Hz and 500-1500Hz): a feature that has been investigated in connection with emotional speech.

Once these features have been extracted. we will apply the Active Data Representation (ADR) method (Haider et al., 2019) for dimensionality reduction, in order to extract the relevant dimensions of variation of the signal, while downplaying contingent voice features. Finally, we will train support vector regression models for automatic prediction of cognitive status (cognitive test scores), and mood, anxiety, and apathy levels (self-reported). We will also explore the relationship between these inferred and self-reported neurobehavioral outcomes and cognitive function through Pearson correlation statistical test and through machine learning modelling, with mood, anxiety and apathy used as additional input features for the model, along with speech features. Where possible, we will discretise the cognitive and neurobehavioral outcomes (mood ratings can be assigned to three classes: depressed, healthy, and (hypo)manic) and train classification models to predict the corresponding classes. Performance of regression models will be assessed throughout the root mean squared error (RMSE) metric and the coefficient of determination. Performance of classification models will be assessed by computing the area under the receiver operating characteristic (ROC) curve, and sensitivity and specificity metrics. We will also generate speech transcripts from the recorded audio and extract word

For peer review only - http://bmjopen.bmj.com/site/about/guidelines.xhtml

embeddings (35) and general linguistic features found to be useful predictors in previous studies, and compare the performance of models built with these features with those based on acoustic features. As we are collecting spontaneous rather than task-based speech, we hypothesise that acoustic features will generalise better to the less constrained setting of our study.

ETHICS AND DISSEMINATION: ETHICAL AND SAFETY CONSIDERATIONS

We consider the main issues to pertain to the remote administration of the study. We have included contact details of the research team on every page of the survey and invite individuals to contact the team should they have any questions after reading the participant information sheet accompanying the informed consent form. We have also engaged with the study sponsor Academic and Clinical Central Office for Research and Development (ACCORD; resgov@accord.scot) and the NHS information governance and IT security teams from the initial design stages of the study protocol to ensure remote consent is a secure method for consent. Our solution was to implement a two-stage consent process whereby the first step of the consent is done entirely remotely but the participant has an option to consent to being contacted about linking to relevant sections in their memory clinic records. If participants consent to being contacted about record linkage, a member of the SIDE-AD study research team contacts the participant and while the second step of the optional record linkage is electronic, it is only after a conversation with a member of the research team which also allows the research team to assess capacity to consent. We note the optional record linkage consent only applies to a small number of participants who are recruited through NHS memory clinics.

We considered this to be an appropriate process from both Good Clinical Practice (GCP) and data security point of view, but this was also the favoured method to consent by our patient and public involvement (PPI) contributors who gave feedback at the design stage of the protocol/survey. The feedback also considered the study to be fairly low impact only taking a few min to complete which does not necessitate face to face consent.

The other issue we have carefully considered is recording individuals' speech. As collecting speech data for clinical research can be difficult due to privacy issues (36), we remind individuals not to disclose personally identifiable information in the speech recording and we also explain that recording of the speech sample is for research purposes only and not part of clinical care.

THE PATIENT AND PUBLIC INVOLVEMENT STATEMENT

Patients and the public were first involved in the research at the design stage. We will also involve patients and public in the analysis of results and dissemination of findings. For the design stage, we contacted the Neuroprogressive and Dementia Network PPI panel in Scotland for feedback on our survey platform, participant information sheet

For peer review only - http://bmjopen.bmj.com/site/about/guidelines.xhtml

and consent forms. We received feedback from 10 individuals both healthy volunteers and those with lived experience of neurodegenerative disease. We carefully reviewed feedback and implemented changes proposed by the PPI contributors, these pertained primarily to the online survey platform but also to the information provided about the study. Overall, the PPI input was favourable, and individuals saw value in a remote study developing speech based biomarkers which was perceived to have low participant burden but reward for taking part in research. Some of the issues raised involved giving more guidance on what people could talk about when they are asked to record speech and to add a box for optional comments for if individuals want to add a note about their recording to the research team. We also intend to involve PPI contributors in the analysis of results and dissemination of findings to help prioritise what information to share, at which conferences/workshops and in which format. We plan to do this via the Neuroprogressive and Dementia Network; EPAD participant panel or National Dementia Carers Action Network (part of Alzheimer Scotland).

DISSEMINATION PLAN

A possible dissemination opportunity will be the Scottish Dementia Research Consortium annual meeting. Additionally, we plan to publish findings from this study in peer-reviewed scientific journals, international conferences such as the AAIC, INTERSPEECH and ICASSP and communicate the study results to participants, healthcare professionals and the public by publishing a lay language report on the university website. We plan to publish a description of the data in venues like Nature Scientific Data and present it at the Language resources and evaluation conference (LREC).

DATA STORAGE

During the study, data will be securely stored on the University of Edinburgh's DataStore service, where it will be accessible only to the research team and not shared outside of the research team in order to protect confidentiality. No personal information about potential participants will be collected as study team will not have access to the individuals' contact details who have been disseminated information about the SIDE-AD study by their parent cohort or NHS clinical teams. Personal information about enrolled participants will be stored securely and separately from research data.

After the study, for longer term preservation of the project's legacy and to ensure reproducibility of our analyses, anonymised research data will be stored on the University of Edinburgh Datashare[1], managed by the University Research Data Service, where the data will be assigned a Digital Object Identifier (DOI). As this project collects voice data, it is not possible to anonymise the data entirely.

[1]https://datashare.ed.ac.uk/handle/10283/8553

For peer review only - http://bmjopen.bmj.com/site/about/guidelines.xhtml

Interested parties can contact the Primary Investigator for request for data and access can be provided on reasonable request with relevant regulatory guidance in place.

ACKNOWLEDGEMENTS

Thank you to the participants enrolled in the SIDE-AD study. We acknowledge Sony Research Awards for their funding of the project and thank the Sony team for their contribution to our thinking around this work.

AUTHOR CONTRIBUTIONS:

SS created a first draft of the protocol with input from SL and FH. SL conceived the idea and design for the study. FH developed the survey software and data management and analysis' plan with input from SS and SL. SS completed the regulatory approvals with input from FH and SL. FH and SS also performed patient and public involvement. SS revised the protocol per suggestions from SL, CWR, GMT. All authors read and approved the final protocol and manuscript for the current paper.

FUNDING STATEMENT: This work was supported by Sony Research Award grant number 11477634- c-00008809.

COMPETING INTERESTS STATEMENT.

Authors declare no competing interests. The funder has no role in the study design; collection, management, analysis or interpretation of data; and in the writing of the report. Because the funder can request that confidential information that belongs to the funder does not get disclosed and that any patentable inventions are protected, the funder is involved in the decision to submit the report for publication. However, if these conditions are met, then the authors have the ultimate authority over the decision to submit the report for publication.

REFERENCES

1. Gauthier S, Rosa-Neto, P., Morais, J.A., Webster, C. Alzheimer's Disease International. World Alzheimer Report. Journey through the diagnosis of dementia. 2021
2. Ballardsych C, Gauthier S, Corbett A, Brayne C, Aarsland D, Jones E. Alzheimer's disease. Lancet (London, England). 2011;377(9770):1019-31.

3. Silva MVF, Loures CdMG, Alves LCV, de Souza LC, Borges KBG, Carvalho MdG. Alzheimer's disease: risk factors and potentially protective measures. Journal of Biomedical Science. 2019;26(1):33.

For peer review only - http://bmjopen.bmj.com/site/about/guidelines.xhtml

4. Ahmad FB, Anderson RN. The Leading Causes of Death in the US for 2020. Jama. 2021;325(18):1829-30.

5. DeTure MA, Dickson DW. The neuropathological diagnosis of Alzheimer's disease. Molecular Neurodegeneration. 2019;14(1):32.

6. McDade E, Wang G, Gordon BA, Hassenstab J, Benzinger TLS, Buckles V, et al. Longitudinal cognitive and biomarker changes in dominantly inherited Alzheimer disease. Neurology. 2018;91(14):e1295-e306.

7. Budd Haeberlein, S., Aisen, P. S., Barkhof, F., Chalkias, S., Chen, T., Cohen, S., Dent, G., Hansson, O., Harrison, K., von Hehn, C., Iwatsubo, T., Mallinckrodt, C., Mummery, C. J., Muralidharan, K. K., Nestorov, I., Nisenbaum, L., Rajagovindan, R., Skordos, L., Tian, Y., van Dyck, C. H., … Sandrock, A. (2022). Two Randomized Phase 3 Studies of Aducanumab in Early Alzheimer's Disease. The journal of prevention of Alzheimer's disease, 9(2), 197–210. https://doi.org/10.14283/jpad.2022.30

8. Rashad A, Rasool A, Shaheryar M, et al. Donanemab for Alzheimer's Disease: A Systematic Review of Clinical Trials. Healthcare (Basel). 2022;11(1):32. Published 2022 Dec 22. doi:10.3390/healthcare11010032

9. Golde TE. Alzheimer's disease – the journey of a healthy brain into organ failure. Molecular Neurodegeneration. 2022;17(1):18.

10. Abdalla M, Rudzicz F, Hirst G. Rhetorical structure and Alzheimer's disease. Aphasiology. 2018;32(1):41-60.

11. Robin, J., Xu, M., Balagopalan, A., Novikova, J., Kahn, L., Oday, A., ... & Teng, E. (2023). Automated detection of progressive speech changes in early Alzheimer's disease. Alzheimer's & Dementia: Diagnosis, Assessment & Disease Monitoring, 15(2), e12445.

12. Williams, E., McAuliffe, M., & Theys, C. (2021). Language changes in Alzheimer's disease: A systematic review of verb processing. Brain and language, 223, 105041. https://doi.org/10.1016/j.bandl.2021.105041

13. Martínez-Nicolás, I., Llorente, T. E., Martínez-Sánchez, F., & Meilán, J. J. G. (2021). Ten Years of Research on Automatic Voice and Speech Analysis of People With Alzheimer's Disease and Mild Cognitive Impairment: A Systematic Review Article. Frontiers in psychology, 12, 620251. https://doi.org/10.3389/fpsyg.2021.620251

For peer review only - http://bmjopen.bmj.com/site/about/guidelines.xhtml

For peer review only

14. De la Fuente Garcia S, Ritchie CW, Luz S. Artificial Intelligence, Speech, and Language Processing Approaches to Monitoring Alzheimer’s Disease: A Systematic Review. Journal of Alzheimer's Disease. 2020;78:1547-74.

15. Posner H, Curiel R, Edgar C, Hendrix S, Liu E, Loewenstein DA, et al. Outcomes Assessment in Clinical Trials of Alzheimer's Disease and its Precursors: Readying for Short-term and Long-term Clinical Trial Needs. Innovations in clinical neuroscience. 2017;14(1-2):22-9.

16. Vrahatis, Aristidis G., Konstantina Skolariki, Marios G. Krokidis, Konstantinos Lazaros, Themis P. Exarchos, and Panagiotis Vlamos. 2023. "Revolutionizing the Early Detection of Alzheimer’s Disease through Non-Invasive Biomarkers: The Role of Artificial Intelligence and Deep Learning" Sensors 23, no. 9: 4184. https://doi.org/10.3390/s23094184

17. Eyigoz E, Mathur S, Santamaria M, Cecchi G, Naylor M. Linguistic markers predict onset of Alzheimer's disease. EClinicalMedicine. 2020;28:100583. Published 2020 Oct 22. doi:10.1016/j.eclinm.2020.100583

18. Brzezińska, A., Bourke, J., Rivera-Hernández, R., Tsolaki, M., Woźniak, J., & Kaźmierski, J. (2020). Depression in Dementia or Dementia in Depression? Systematic Review of Studies and Hypotheses. Current Alzheimer research, 17(1), 16–28. https://doi.org/10.2174/1567205017666200217104114

19. Hajjar, I., Okafor, M., Choi, J. D., Moore, E., 2nd, Abrol, A., Calhoun, V. D., & Goldstein, F. C. (2023). Development of digital voice biomarkers and associations with cognition, cerebrospinal biomarkers, and neural representation in early Alzheimer's disease. Alzheimer's & dementia (Amsterdam, Netherlands), 15(1), e12393. https://doi.org/10.1002/dad2.12393

20. Lim YY, Maruff P, Pietrzak RH, Ames D, Ellis KA, Harrington K, et al. Effect of amyloid on memory and non-memory decline from preclinical to clinical Alzheimer's disease. Brain. 2014;137(Pt 1):221-31.

21. Boschi, V., Catricala, E., Consonni, M., Chesi, C., Moro, A., & Cappa, S. F. (2017). Connected speech in neurodegenerative language disorders: a review. Frontiers in psychology, 8, 269.

For peer review only - http://bmjopen.bmj.com/site/about/guidelines.xhtml

22. Öhman F, Hassenstab J, Berron D, Schöll M, Papp KV. Current advances in digital cognitive assessment for preclinical Alzheimer's disease. Alzheimer's & dementia (Amsterdam, Netherlands). 2021;13(1):e12217-e.

23. Luz S, Haider F, Fromm D, MacWhinney B, (eds.). Alzheimer's Dementia Recognition Through Spontaneous Speech. Lausanne, Switzerland: Frontiers Media S.A., 2021. 258 p.

24. de Lope, J., Graña, M. 2023. An ongoing review of speech emotion recognition. Neurocomputing. 528 (1-11) https://doi.org/10.1016/j.neucom.2023.01.002

25. Landes AM, Sperry SD, Strauss ME. Prevalence of apathy, dysphoria, and depression in relation to dementia severity in Alzheimer's disease. The Journal of neuropsychiatry and clinical neurosciences. 2005 Aug;17(3):342-9.

26. Babulal GM, Ghoshal N, Head D, Vernon EK, Holtzman DM, Benzinger TL, Fagan AM, Morris JC, Roe CM. Mood changes in cognitively normal older adults are linked to Alzheimer disease biomarker levels. The American journal of geriatric psychiatry. 2016 Nov 1;24(11):1095-104.

27. Ramanarayanan, V., Lammert, A. C., Rowe, H. P., Quatieri, T. F., & Green, J. R. (2022). Speech as a biomarker: Opportunities, interpretability, and challenges. Perspectives of the ASHA Special Interest Groups, 7(1), 276-283.

28. Luz S. Longitudinal Monitoring and Detection of Alzheimer's Type Dementia from Spontaneous Speech Data. In Computer Based Medical Systems. Institute of Electrical and Electronics Engineers (IEEE). 2017. p. 45-46.

29. Ritchie, C. W., & Ritchie, K. (2012). The PREVENT study: a prospective cohort study to identify mid-life biomarkers of late-onset Alzheimer's disease. BMJ open, 2(6), e001893. https://doi.org/10.1136/bmjopen-2012-001893

30. Riley RD, Ensor J, Snell KI, Harrell FE, Martin GP, Reitsma JB, Moons KG, Collins G, Van Smeden M. Calculating the sample size required for developing a clinical prediction model. Bmj. 2020 Mar 18;368.

31. Raudys SJ, Jain AK. Small sample size effects in statistical pattern recognition: recommendations for practitioners. IEEE Trans PatternAnal Mach Intell 1991;13:252–64.

For peer review only - http://bmjopen.bmj.com/site/about/guidelines.xhtml

32. Eyben F, Scherer KR, Schuller BW, et al. The Geneva Minimalistic Acoustic Parameter Set (GeMAPS) for voice research and affective computing. IEEE Trans Affect Comput 2016;7:190–202.

33. De la Fuente Garcia S, Haider F, Luz S. COVID-19: Affect recognition through voice analysis during the winter lockdown in Scotland. In2021 43rd Annual International Conference of the IEEE Engineering in Medicine & Biology Society (EMBC) 2021 Nov 1 (pp. 2326-2329). IEEE.

34. Haider, F., De La Fuente, S. and Luz, S., 2019. An assessment of paralinguistic acoustic features for detection of Alzheimer's dementia in spontaneous speech. IEEE Journal of Selected Topics in Signal Processing, 14(2), pp.272-281.

35. Devlin, J., Chang, M.W., Lee, K. and Toutanova, K., 2018. Bert: Pre-training of deep bidirectional transformers for language understanding. arXiv preprint arXiv:1810.04805.

36. Voleti, R., Liss, J. M., & Berisha, V. (2019). A review of automated speech and language features for assessment of cognitive and thought disorders. IEEE journal of selected topics in signal processing, 14(2), 282-298.

For peer review only

For peer review only - http://bmjopen.bmj.com/site/about/guidelines.xhtml

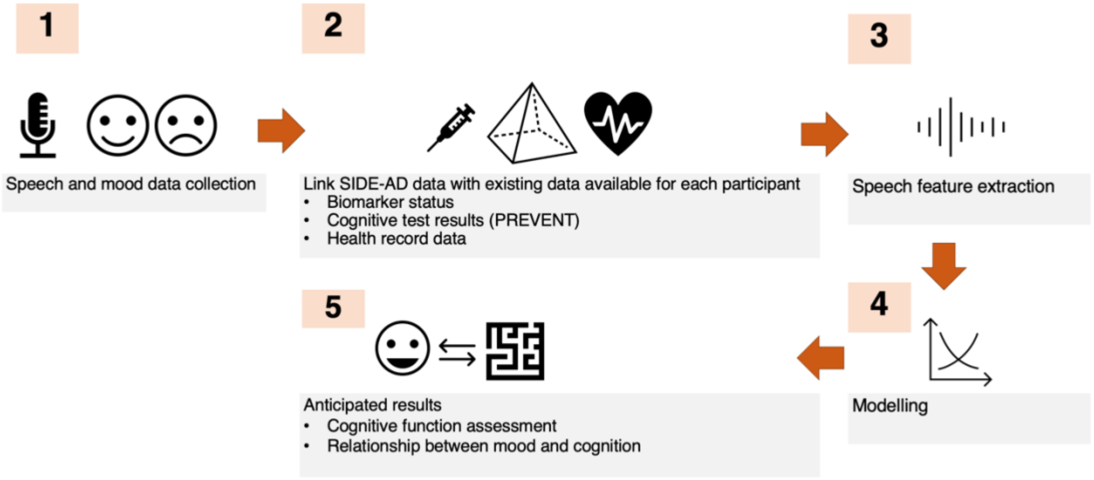

For peer review only

THE UNIVERSITY of EDINBURGH

NHS Lothian

Participant Information Sheet

Speech for Intelligent cognition change tracking and DEtection of Alzheimer's Disease Research Program: SIDE-AD

You are being invited to take part in a research study. Before you decide whether or not to take part, please take time to read the following information carefully. Talk to your family about the study if you wish.

Chief Investigator:
Saturnino Luz, PhD

Research team address:
University of Edinburgh
Usher Institute of Public Health
9 Little France Road
Edinburgh BioQuarter
EH16 4UX
side-ad@ed.ac.uk

For peer review only

What is the purpose of the study?

Each year we understand more about brain health and how this relates to dementia. Although the brain changes for all of us as we age, and many changes are normal, abnormal changes can be due to an underlying brain disease. One of the most common brain diseases is Alzheimer's disease, which causes dementia.

We now know that when Alzheimer's disease develops, there are changes in the brain which may occur many years before memory and thinking skills are affected. This is typically many years before somebody is ultimately diagnosed with dementia. Throughout this information sheet, when we talk about Alzheimer's disease, we mean the underlying brain disease that over many years may lead to Alzheimer's dementia. Currently, doctors can only diagnose Alzheimer's disease at the most advanced stage when people already have dementia or are very close to developing it.

The purpose of this study is to understand more about speech as potential early indicator of dementia risk. This is because changes in how somebody speaks may show markers of pathological changes in Alzheimer's disease. The SIDE-AD study is a

For peer review only - http://bmjopen.bmj.com/site/about/guidelines.xhtml

longitudinal cohort study which means that if you take part in the study, you will be emailed a follow-up email to carry out follow-up assessments. Taking part in the study and in the follow-up assessments is all voluntary.

This study aims to develop digital speech-based markers for Alzheimer's disease. Please note that the speech assessment is not a diagnostic tool for Alzheimer's disease but is used to study the disease.

Why have I been invited to take part?

You have been approached because you are either healthy and do not experience cognitive decline or you are engaged with secondary services for cognitive decline and may or may not have a diagnosis related to memory concerns.

We invite individuals through the following paths:

- Taking part in the PREVENT cohort study
- Being a member of a research register
- Attending NHS memory assessment services

We aim to recruit 350 participants from the PREVENT cohort study and research registers and 150 participants from NHS services.

To find out which factors may predict risk of Alzheimer's dementia, we need a broad range of people with different likelihoods of developing Alzheimer's dementia. We do not know in advance who will develop dementia and who will not. This means we aim to include in the SIDE-AD study people who are possibly at high risk, low risk and anywhere in between. As we follow all the people in the SIDE-AD study, we learn which factors predict risk of dementia. Only by including a wide range of people can we identify risk factors Alzheimer's dementia.

Research team address:
University of Edinburgh
Usher Institute of Public Health
9 Little France Road
Edinburgh BioQuarter
EH16 4UX; side-ad@ed.ac.uk

For peer review only - http://bmjopen.bmj.com/site/about/guidelines.xhtml

THE UNIVERSITY of EDINBURGH

NHS Lothian

Do I have to take part?

No, it is up to you to decide whether or not to take part. If you decide to take part, you are still free to withdraw at any time and without giving a reason. Deciding not to take part or withdrawing from the study will not affect the clinical care that you receive.

What will happen if I take part?

This is a longitudinal study. Longitudinal simply means that we will send out emails for follow-up assessments every three months over two years. All assessments are done remotely at home, and you are free to withdraw at any point. Each study assessment will take about 5 min and you are asked to use either your computer or any smart device. A study assessment includes:

- *Background information about you*
- *Four questions about your mood, anxiety and apathy levels*
- *Recording yourself speak for about a minute for speech analysis*
  You could talk about any aspects about brain health. For example, you could talk about what brain health means to you, what you do to look after your brain health or what you feel you could do differently.

If you decide to take part in the study, please go to the below website:

tiny.cc/speechstudy

Before you start the study, we ask that you read this information sheet and consent to taking part in the study. Please read the statements carefully and write your initials in the box if you agree. If you have any questions about the study, please contact the research team. After you have given electronic consent to taking part in the study, the assessments will start.

Research team address:
University of Edinburgh
Usher Institute of Public Health
9 Little France Road
Edinburgh BioQuarter
EH16 4UX; side-ad@ed.ac.uk

For peer review only - http://bmjopen.bmj.com/site/about/guidelines.xhtml

THE UNIVERSITY of EDINBURGH

NHS Lothian

Please note that on the consent form, we will also ask for your permission to be contacted to discuss record linkage. If you consent to this, a member of our research team will contact you and have an informed discussion with you whether you agree to record linkage and take your consent. Record linkage is applicable for participants who are recruited through clinical services and means that we can match your study data to your health records collected during your routine NHS care. We will do this using your name and date of birth. This can be really useful to researchers to understand what research data means in relation to clinical findings such as brain scans, routine blood tests and treatment decisions or diagnosis. You do not have to agree to this to be in the study.

if you are having problems completing the study activities during the course of the study (meaning you have difficulties understanding information and making a decision based on it), you can still remain in the study unless you decide to leave. This simply means that whether you experience problems with your brain health such as remembering things and planning or you do not experience these kinds of problems, you are welcome to continue taking part in our study. if you are having problems completing the study activities, you are welcome to get help from friends or family with any aspects of the study.

At any stage in the study, if you no longer wish to continue, you can withdraw from either all aspects of the study but allow us to use data collected up to the point where you discontinue **OR** you can withdraw from all aspects of the study with removal of all previously collected data. You simply have to tell us what you prefer. It is important to realise that when you start the study you are free to leave at any time point after giving consent.

Research team address:
University of Edinburgh
Usher Institute of Public Health
9 Little France Road
Edinburgh BioQuarter
EH16 4UX; side-ad@ed.ac.uk

For peer review only - http://bmjopen.bmj.com/site/about/guidelines.xhtml

THE UNIVERSITY of EDINBURGH

NHS Lothian

What are the possible benefits of taking part?

You should not expect to benefit medically from being involved in this project. Participants can find participating in studies like this enjoyable.

Your contribution to this research enables us to further our understanding of Alzheimer's disease. Specifically, taking part in the study may help researchers and clinicians in identifying early signs of Alzheimer's disease and ultimately to treat or prevent dementia.

What are the possible disadvantages of taking part?

We appreciate that taking part in the study assessments is a time commitment and we will do everything we can to fit into your schedule and be as flexible as possible.

The study is entirely remote which means you can choose the time when is suitable for you to do the study assessments.

What if there are any problems?

If you have a concern about any aspect of this study, please contact the study team who will be delighted to answer any questions about the study. If you would like to discuss this study with someone independent of the study, please contact Dr Miles Welstead (Miles.Welstead@ed.ac.uk) who will do his best to answer your questions. However, if you are still unhappy with the answers given and wish to complain, you can do this through the NHS Lothian Patient Experience Team (2nd Floor; Waverley Gate; 2 – 4 Waterloo Place; Edinburgh; EH1 3EG; Tel: 0131 536 3370; email: feedback@nhslothian.scot.nhs.uk).

Will my taking part be kept confidential?

You will be assigned a participant number and you will not be personally identified in any of the research data. Your personal data, including your voice recordings, will not leave the research site. Your voice data will be stored on secure

Research team address:
University of Edinburgh
Usher Institute of Public Health
9 Little France Road
Edinburgh BioQuarter
EH16 4UX; side-ad@ed.ac.uk

For peer review only - http://bmjopen.bmj.com/site/about/guidelines.xhtml

THE UNIVERSITY of EDINBURGH

NHS Lothian

University of Edinburgh servers (servers are large storage spaces for computer data and be password protected) and will only be accessible to legitimate members of the research team and is approved by the sponsor. In order to monitor and audit the study we will ask for your consent for responsible representatives from the study sponsor and NHS Institution(s) to access your data collected during the study. The Sponsors are responsible for overall management of the study and providing insurance and indemnity. The sponsor is the University of Edinburgh and NHS Lothian under a joint agreement between those two organisations called ACCORD.

What will happen to the results of the study?

To analyse early indicators of Alzheimer's disease, we pool together data from all the study participants. This means that we do not analyse individual participant trajectories (changes in risk factors over time) but look for patterns across all the study participants.

External researchers will be able to apply to access research data from the SIDE-AD study. Please note that your voice recordings will not be shared. Research data is anonymous, and you will not be identifiable in any of this data. We think this is important to maximise the use of all data collected in the study. Sony Research Awards who funds this study may have access to the anonymised results of this study.

Your voice data may be transcribed by an approved 3rd party vendor. This means that the transcribing company we use will respect the confidentiality of your data and their work has been approved by the University of Edinburgh. Transcribing means writing out all the spoken information you give and we record into text. All data transcripts will be anonymised. We will prepare scientific publications based on the findings from the SIDE-AD study which will be presented at national and international conferences. You will not be identified in any of the publications. We will communicate findings from these studies in regular newsletters, which you can sign up to receive.

Research team address:
University of Edinburgh
Usher Institute of Public Health
9 Little France Road
Edinburgh BioQuarter
EH16 4UX; side-ad@ed.ac.uk

For peer review only - http://bmjopen.bmj.com/site/about/guidelines.xhtml

THE UNIVERSITY of EDINBURGH

NHS Lothian

Where can you find out more about how your information is used?

You can find out more about how we use your information

- at www.hra.nhs.uk/information-about-patients/
- our leaflet available from www.hra.nhs.uk/patientdataandresearch
- by asking one of the research team
- by sending an email to the Sponsor's Data Protection Officer dpo@ed.ac.uk

Who is organising and funding the research?

This study is led by the University of Edinburgh, Dr Saturnino Luz. The other team members include Dr Fasih Haider and Dr Stina Saunders who are both postdoctoral research fellows at the University of Edinburgh. This study is funded by Sony Research Awards.

Who has reviewed the study?

All research in the NHS is looked at by an independent group of people called a Research Ethics Committee. A favourable ethical opinion has been obtained from XXXX. NHS management approval has also been given. IRAS Project ID 323311.

Researcher Contact Details

For any questions concerns, please email Dr Stina Saunders (Stina.Saunders@ed.ac.uk) or dedicated study contact email side-ad@ed.ac.uk.
University of Edinburgh
Usher Institute of Public Health
9 Little France Road
Edinburgh BioQuarter
Edinburgh
EH16 4UX

Independent Contact Details

If you would like to discuss this study with someone independent of the study, please contact Dr Miles Welstead (Miles.Welstead@ed.ac.uk)

Complaints

If you wish to make a complaint about the study please contact:

Patient Experience Team
2 – 4 Waterloo Place, Edinburgh, EH1 3EG
feedback@nhslothian.scot.nhs.uk
0131 536 3370

Thank you for considering your involvement in this project and taking time to read this information sheet

For peer review only - http://bmjopen.bmj.com/site/about/guidelines.xhtml

Electronic Informed Consent Form for Participants

Speech for Intelligent cognition change tracking and DEtection of AD (SIDE-AD) Research Program: SIDE-AD

This document is for review purposes only, consent takes place electronically using the following link tiny.cc/speechstudy

	Enter your Initials (electronic)
1. I confirm that I have read and understand the information sheet (SIDE-AD PIS v4 29 Nov 2023) for the above study. I have had the opportunity to consider the information, ask questions and have had these questions answered satisfactorily.	
2. I understand that my participation is voluntary and that I am free to withdraw at any time without giving any reason and without my medical care and/or legal rights being affected.	
3. I understand that data collected about me during the study may be converted to anonymised data.	
4. I understand that relevant sections of my data collected during the study may be looked at by individuals from the Sponsor (University of Edinburgh and NHS Lothian under a joint agreement between those two organisations called ACCORD) and/or from regulatory authorities where it is relevant to my taking part in this research. I give permission for these individuals to have access to my data and/or medical records.	
5. I agree to my voice being recorded as part of the study.	
6. I understand that if I lose capacity during the course of this study, I will remain in the current study or I am free to withdraw. This means that whether I experience problems with my brain health or I do not experience problems, I am welcome to continue taking part in this study.	

SIDE-AD ICF v1 24 Jul 2023
IRAS Project ID: 323311

For peer review only - http://bmjopen.bmj.com/site/about/guidelines.xhtml

THE UNIVERSITY of EDINBURGH

NHS Lothian

7. I agree to my audio recordings being transcribed by a third-party contractor.	
8. I understand that information collected will be managed by the research team only and will be destroyed after a period of ten years and any information collected will be kept in line with the data protection act in a secure way.	
9. I agree to my anonymised data being used in future studies.	
10. I agree to be re-contacted about future research.	
11. I confirm that I am over 40 years of age.	
12. I agree to take part in the above study.	
Final statement is only applicable to individuals recruited through NHS memory clinics. *If you agree to this statement, a member of the research team will contact you to explain this further and ask for your consent to access relevant sections of your Medical Records.* *Agreeing to this statement* ***does not grant access*** *to your Medical Records. Please enter* ***initials*** *below if you agree.*	
**13. Optional:** I agree to be contacted about the potential to link my study data with my NHS Medical Records.	
Enter Name:	
Date:	

For peer review only

For peer review only - http://bmjopen.bmj.com/site/about/guidelines.xhtml
